# ERYXSeg: a hybrid CNN architecture for robust and resource-aware wound segmentation

**DOI:** 10.3389/fsurg.2026.1790725

**Published:** 2026-06-19

**Authors:** Trishaani Acharjee, Rajdeep Chatterjee, Mahendra Kumar Gourisaria, Manoj Sahni, Ernesto León-Castro

**Affiliations:** 1 School of Computer Engineering, KIIT Deemed to be University, Bhubaneswar, India; 2 Department of Mathematics, School of Technology, Pandit Deendayal Energy University, Gandhinagar, Gujarat, India; 3 Faculty of Economics and Administrative Sciences, Universidad Católica de la Santísima Concepción, Concepción, Chile

**Keywords:** deep learning, foot ulcer, image segmentation, medical imaging, wounds

## Abstract

In modern clinical practice, the accuracy of wound segmentation is important because it allows for data-driven treatment planning, accurate evaluation of the wound area, and tracking of the healing process. In this paper, we introduce ERYXSeg, an integrated deep learning architecture that combines residual skip connections, a parameter-efficient core, and spatial localization and boundary-aware refinement of a segmentation head. The proposed method achieves robust performance across a wide range of wound types by capturing both local fine-grained characteristics and rich contextual information. ERYXSeg outperforms the current state-of-the-art models under identical training settings after being trained from scratch on mixed wound datasets. It achieves the highest reported IoU and Dice scores in both datasets: 0.7633 IoU and 0.8658 Dice for the foot ulcer dataset and 0.6910 IoU and 0.8173 Dice for the curated mixed wound dataset. The contribution of each architectural element is further validated by a systematic ablation study, which shows that attention-gated skip connections are crucial for accurate spatial reconstruction across various data sources and that Mobile Inverted Bottleneck Convolution (MBConv) blocks are essential for extracting broadly applicable features. The results obtained demonstrate excellent generalization and high computational efficiency of the model, which thus qualifies it for real-time clinical deployment independent of transfer learning.

## 1 Introduction

Modern diagnostics rely considerably on medical image analysis, which provides data-driven insights that go beyond the constraints of conventional subjective evaluation. While deep learning has fully transformed the methods of approach for radiology and pathology images, wound image analysis remains relatively unexplored. This is a critical gap because accurate wound segmentation and analysis are essential for measuring tissue viability, monitoring the healing process, and developing successful treatment strategies for serious illnesses such as pressure ulcers and diabetic foot ulcers. The manual evaluation in clinical use is time-consuming and undergoes a high degree of variation due to human interference. This makes consistent and scalable patient monitoring difficult. These challenges constitute a critical call for reliable, automated systems that can produce pixel-level measurements. The automated and accurate analysis of wound images thus constitutes an important and influential problem in clinical treatment, with direct consequences for patient outcomes. The accurate and automatic analysis of wound images remains a significant challenge in medical care, directly impacting patient welfare. Chronic wounds, such as diabetic foot ulcers and pressure wounds, require close monitoring to track the healing process, to avoid complications such as infection, and to create a more effective treatment plan [1–4].

Early attempts at wound analysis used basic image processing algorithms. Techniques such as color-based thresholding, edge detection filters, and region growing approaches were considered innovative in their time. However, they are not very efficient in practical scenarios. Common factors such as uneven lighting, a wide range of backgrounds, and the sometimes complicated and irregular textures of various types of wounds all significantly impact the processing of images. The recent development of deep learning, particularly CNNs, has radically changed this paradigm. CNNs have shown far greater capability to handle the complexity of images in practical situations, such as learning hierarchical features directly from data and attaining diagnostic performance; they offer a helping hand to the medical personnel (5–9).

In this domain, wound segmentation is one of the most important tasks. Unlike image classification, which involves assigning one label to an entire image, segmentation provides a pixel-by-pixel classification that helps highlight the exact edge of the wound. This can be very useful in calculating the surface area, measuring depth contours, and tracking minute changes in the structure of the wounds over time, which is beneficial for detailed clinical analysis [10–15].

However, several challenges stand in the way of developing robust segmentation models, including processing costs, intrinsic heterogeneity, and a lack of annotated data. It takes a significant amount of time to annotate a dataset at the pixel level, requiring extensive involvement of specialized medical knowledge. As a result, very few public benchmark datasets exist. Color, bloody skin, surrounding tissue, and shape all vary greatly, giving every wound a very distinct appearance. Therefore, the model’s generalization must be very high. The structures of high-accuracy models are often complex and resource-intensive, which is problematic for real-time deployment.

In this work, we introduce ERYXSeg, a novel deep learning architecture for both accuracy and efficiency. The key benefits arise from multi-scale feature extraction with EfficientNet, strong gradient flow with residual connections from ResNet, and sophisticated real-time instance segmentation from the YOLOv8 framework, all fully integrated into one model: ERYXSeg. This synergistic design aims to balance computational viability with highly accurate boundary detection.

Our work is motivated by several gaps that have been identified in the state of automated wound analysis. First, current approaches frequently rely heavily on transfer learning from non-medical domains, which may limit model generalization to the distinctive textural and colorimetric characteristics of wound tissue. In contrast, through our work, we have shown that training from scratch on specific data can also yield the desired results. Second, a lot of research in this field is primarily carried out on a single dataset, such as the diabetic foot ulcer (DFU) dataset, or on data that are private due to confidentiality, indicating a lack of data availability and diversity. This makes it difficult to assess the true robustness of models across a variety of wound types and imaging conditions. Lastly, more thorough evaluation techniques are required. Although standard metrics are reported in much of the research, unsuccessful case analysis and detailed qualitative verification are inadequate. Our work aims to address these gaps by introducing a model trained from scratch that undergoes rigorous evaluation on multiple datasets, further supported by in-depth visualization to validate its use in the medical field. In the medical context, a segmentation model must satisfy two essential and often contradictory needs. When incorporated into real-world applications, such as a clinical device with limited resources or a mobile healthcare application, the model must be computationally lightweight to enable rapid inference without compromising accuracy; in medical diagnostics, accuracy is the top priority. To provide an architecture that is both extremely accurate and sufficiently efficient for real-world deployment, ERYXSeg’s design aims to navigate this important trade-off.

We have used some crucial preprocessing and augmentation steps to overcome the class imbalance while making the model more robust in nature. This work offers not only a robust model for wound assessment but also indicates the necessity for scalable and effective deep learning solutions in medicine, taking into account the architectural and data-centric obstacles. The organization of the rest of this paper is as follows: Section 2 presents related work in medical image segmentation. Section 3 describes the source of data and the preprocessing methodology that has been followed. The ERYXSeg architecture is presented in depth in Section 4. Section 5 describes the experimental design and metrics for performance evaluation, followed by an elaborate presentation and discussion of the results in Section 6. Finally, concluding remarks and future directions for research are presented in Section 7.

## 2 Related work

Recent literature has demonstrated the transformative role of AI in wound image analysis. In the following, we group key contributions by methodology:

Scebba et al. [16] introduce a two-stage deep learning pipeline. Their method first detects the wound using a RetinaNet detector and then segments it with a UNet. Trained on datasets such as DFUC 2020 and SwissWOU-DFU, with augmentations including flipping and brightness changes, this approach achieved robust results (MCC: 0.85) and strong generalization across various wound types and imaging devices. A noted limitation is the model’s potential failure in the initial detection stage and a lack of ethnic diversity in the training data. Cui et al. [17] utilize a dataset of 445 images from NYU. Their preprocessing involved white spot removal and color constancy normalization. They implemented and compared a patch-based CNN and a UNet architecture, with the UNet achieving superior results (Dice: 0.845). The model’s primary limitation is its lack of generalization testing on other imaging devices or resolutions, as it is trained and evaluated on a single pre-cropped dataset. Ramachandram et al. [18] has used the large, diverse Swift Wound Data Set. They implemented mobile-optimized encoder-decoder CNNs (AutoTrace and AutoTissue), which achieved a mean IoU of 0.7192 for segmenting granulation, slough, and eschar tissues. A key limitation is the model’s poor performance in segmenting epithelial tissue, attributed to its underrepresentation in the training data and high labeling variability among clinicians. Wang et al. [19] has utilized a proprietary dataset of 1,109 foot ulcer images. Their preprocessing involves wound localization using YOLOv3 and cropping. They implemented a MobileNetV2-based encoder-decoder model, achieving a high Dice score of 90.47%. A key limitation of this efficient model is that its performance and generalizability to other wound types beyond foot ulcers have not been fully established. Oota et al. [20] introduces the large, diverse WOUNDSEG dataset. Their method employed a global-local CNN architecture with wound-domain adaptive pre-training and data augmentation, achieving a state-of-the-art Dice score of 0.847. A key limitation is the computational complexity of the two-stream architecture, which may hinder deployment on resource-constrained devices despite its high accuracy. Wang et al. [21] propose a public dataset of 1,210 foot ulcer images. The winning model is an ensemble of UNet and LinkNet with EfficientNet backbones, utilizing data augmentation and post-processing to achieve a top Dice score of 88.80%. A key limitation is the high computational cost and inference time of the ensemble method, which may be impractical for real-time clinical deployment. A short overview is presented in the form of a table below (refer to Table 1).

**Table 1:** Comparative summary of wound segmentation research papers.

Paper name	Author(s)	Dataset used	Model used	Result achieved
Detect-and-Segment: A Deep Learning Approach to Automate Wound Image Segmentation	Scebba et al. [16]	DFUC 2020, SwissWOU (SW-DFU, SW-SSD), Medtec, SIH, FUSC	**Pipeline:** RetinaNet (MobileNet) Detector + UNet Segmenter	**MCC:** 0.85, **IoU:** 0.75 (Best with DS approach on SW-DFU)
Diabetic Wound Segmentation using Convolutional Neural Networks	Cui et al. [17]	445 images from NYU	**Models:** Patch-based CNN, UNet	**Dice:** 0.845 (UNet with post-processing)
Fully Automated Wound Tissue Segmentation Using Deep Learning on Mobile Devices	Ramachandram et al. [18]	Swift Wound Data Set (˜465k wound, 17k tissue labels)	**Models:** AutoTrace & AutoTissue (Mobile-optimized Encoder-Decoder CNNs)	**IoU:** 0.7192 (Tissue segmentation); Poor epithelial tissue segmentation
Fully automatic wound segmentation with deep convolutional neural networks	Wang et al. [19]	Proprietary dataset of 1,109 foot ulcer images	**Model:** MobileNetV2 Encoder-Decoder	**Dice:** 90.47% (with post-processing)
WSNet: Towards An Effective Method for Wound Image Segmentation	Oota et al. [20]	WOUNDSEG (2,686 images, 8 wound types)	**Model:** Global-Local CNN (e.g., LinkNet with DenseNet) + Wound-Domain Pretraining	**Dice:** 0.847 (State-of-the-art on WOUNDSEG)
FUSeg: The Foot Ulcer Segmentation Challenge	Wang et al. [21]	FUSeg (1,210 foot ulcer images)	**Winning Model:** Ensemble of UNet (EfficientNetB2) & LinkNet (EfficientNetB1)	**Dice:** 88.80% (Top score on challenge test set)

## 3 Methodology

### 3.1 The dataset

The proposed segmentation methodology uses two different datasets.

#### 3.1.1 Foot ulcer dataset

**Figure 1:**
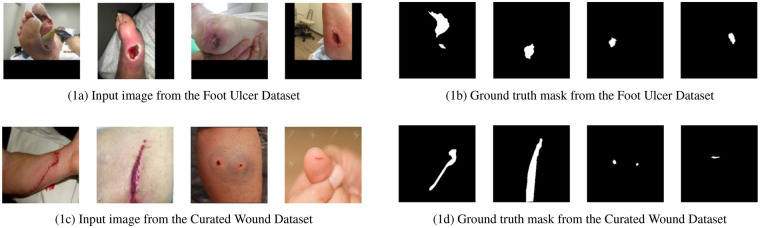
Visualization of the datasets used in this study: **(a)** Sample image from the Foot Ulcer dataset; **(b)** corresponding ground truth mask; **(c)** sample image from the curated Wound dataset; **(d)** corresponding wound mask.

The main dataset used in this work consists of foot ulcer wound images obtained from GitHub^1^ [22]. Considering the clinical importance of foot ulcers in diabetic patients due to the high risk of infection and possible amputation, the dataset has established a clear benchmark for the segmentation analysis of wounds. It contains a total of 1,109 RGB images collected from 889 patients, along with corresponding ground truth masks that outline ulcer regions, as shown in Figures 1a,b. The RGB images are originally in .jpg format, whereas the masks are provided in binary .png format, with 0 pixel values for background skin and 1 for ulcerous areas. The entire dataset is split into an 80% (567 images) training set, 10% (121 images) for validation, and 10% (122 images) for testing.

#### 3.1.2 Curated wound dataset

To further evaluate the generalization capability of the proposed approach, a secondary curated dataset consisting of 1,500 wound images is constructed using images from Kaggle^2^ [23]. The ground truth masks for this dataset are carefully created using Roboflow, ensuring accurate annotation of wound boundaries (shown in Figures 1c,d). Annotated polygons are converted from the original wound images into their binary segmentation masks, where wound regions are distinctly labeled from the surrounding tissue. The curated wound dataset includes various types of wounds and anatomical locations, providing a complementary test set to assess the model’s robustness across different presentations of wounds. Similar to the primary dataset in this work, this dataset maintains the same data split ratio 80% for training, 10% for validation, and 10% for testing, thus allowing for consistent comparative analysis. Both datasets have passed through identical preprocessing pipelines and augmentation strategies to maintain consistency in the experiments.

The entire methodology is implemented using Python 3.10.0 in the PyTorch environment, along with Torch 2.5.1, with *torch.amp enabling automatic mixed precision training, which accelerates deep learning by using both float16 and float32 where appropriate*, and CUDA 12.1 for efficient computation. Adding these two datasets will allow for a comprehensive assessment of the model’s performance on specialist foot ulcer segmentation, as well as more generalized wound analysis.

We have trained and assessed ERYXSeg on two different datasets to guaranty the wide clinical applicability of our model. The first dataset is the well-known diabetic foot ulcer (DFU) dataset, which provides a standard against which contemporary state-of-the-art models can be compared. The second is a carefully chosen mixed wound dataset that includes cuts, lacerations, abrasions, and surgical wounds. This choice is motivated by the realities of medical practice, where common acute wounds occur far more frequently than chronic ulcers. We have intentionally constructed our model to generalize beyond a single wound type by embracing this diversity to develop a trustworthy model that can segment a wide variety of wound images observed in everyday medical treatment.

#### 3.1.3 Inter-annotator agreement analysis

Cohen’s Kappa coefficient (κ) was used as a statistical measure of inter-annotator agreement to evaluate the consistency and dependability of the manual annotation process. Cohen’s Kappa is a more reliable metric than simple percentage agreement because it measures the degree of agreement between two annotators while taking into consideration the possibility of agreement occurring by chance. Almost perfect agreement is indicated by values above 0.81, substantial agreement is indicated by values between 0.61 and 0.80, moderate agreement is indicated by values between 0.41 and 0.60, fair agreement is indicated by values between 0.21 and 0.40, and slight or poor agreement is indicated by values below 0.20.

**Figure 2:**
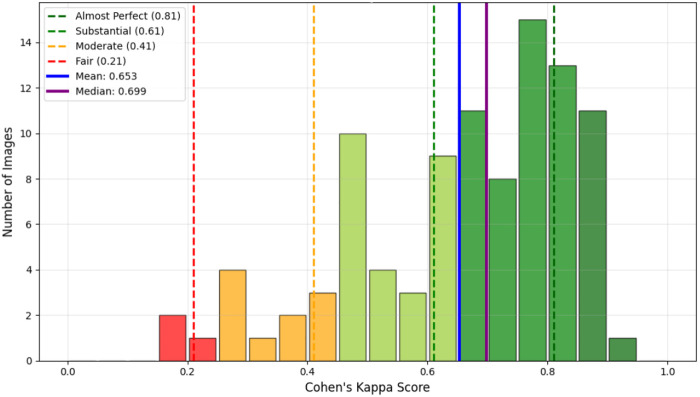
Distribution of Cohen’s Kappa scores (κ) for inter-annotator agreement. Mean κ=0.653 indicates substantial agreement, validating the reliability of the ground truth annotations.

A second independent annotator was hired to manually annotate the same set of wound images in order to assess the annotation quality of the curated wound dataset. The agreement between the original and the second annotator’s segmentation masks was then calculated using Cohen’s Kappa. With a mean Kappa score of 0.653, the distribution of Kappa scores throughout the dataset shows substantial to nearly perfect agreement for most images, as shown in Figure 2. Just 8% of the images fell below the moderate agreement threshold, whereas about 68% of the images had Kappa scores above 0.61, indicating significant agreement between annotators. The reliability of the ground truth annotations and the caliber of the carefully selected dataset for segmentation tasks are both confirmed by this high degree of inter-annotator consistency. For training reliable deep learning models and guaranteeing the validity of comparative performance assessments, the Kappa distribution analysis offers assurance that the annotations are repeatable and consistent.

Even though a Kappa value of 0.653 might seem moderate-to-substantial, it’s crucial to consider the unique difficulties associated with wound segmentation. Because even slight edge differences can have a substantial impact on Kappa while maintaining semantic agreement, pixel-wise segmentation naturally magnifies small boundary deviations between annotators. This is especially important for wound areas, where biological and visual ambiguity make boundary uncertainty inevitable. Precise boundary delineation is intrinsically difficult because wound margins are frequently irregular, with gradual transitions between necrotic tissue and healthy skin. Numerous pixel mismatches that artificially lower the Kappa coefficient can occur when two annotators agree on the overall wound region but differ slightly at the edges, one may include thin peripheral pixels while another excludes them. This phenomenon is well known in the field of medical image segmentation, where Kappa is frequently used in conjunction with overlap-based metrics like Dice and IoU to provide a more thorough evaluation of segmentation quality. The high level of inter-annotator consistency found in this study validates the quality of the curated dataset for segmentation tasks and confirms the accuracy of the ground truth annotations, especially in light of these inherent annotation challenges. The Kappa distribution analysis gives assurance that the annotations are repeatable and consistent, which is useful for training strong deep learning models and guaranteeing the validity of comparative performance evaluations.

### 3.2 Preprocessing the images

Several preprocessing steps are applied to ensure consistency, enhance generalization, and maximize learning efficiency before providing the input to the model. Each image and its corresponding mask are resized to a spatial dimension of 256×256 to maintain consistency throughout the dataset while avoiding excessive computational costs and still retaining important spatial information. Geometric transformations include random horizontal and vertical flips to further increase variety and reduce overfitting. Each flip is performed with a probability of 0.5. Normalizing the images, which means dividing by 255, scales the pixel values to the range between [0,1]. In this regard, normalization is crucial for stabilizing training, as it brings values to a comparable scale and reduces the impact of large pixel intensities. The masks, which represent segmentation labels, are also scaled and transformed into tensors while maintaining class indices. These combined techniques enhance the model’s ability to generalize on previously unseen wound images and allow for robust training.

### 3.3 Skip connections for feature preservation

**Figure 3:**
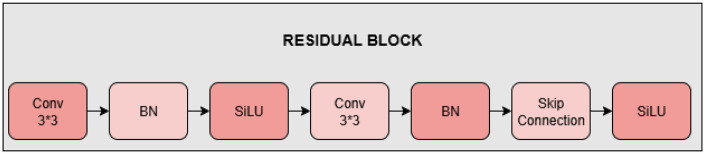
Architectural components derived from ResNet integrated within the ERYXSeg framework.

Residual Networks (ResNets) introduce a powerful architectural paradigm through the use of identity skip connections, which facilitate residual learning. This framework reformulates the underlying learning objective for a stack of layers: rather than directly approximating a complex underlying mapping H(x), the network learns the residual function F(x)=H(x)−x. The final output is then given by F(x)+x. This mechanism significantly improves the vanishing gradient problem in very deep networks by creating open pathways for gradient flow during backpropagation. Within the ERYXSeg architecture, we integrate ResNet-style residual blocks to construct a robust feature hierarchy. These blocks are essential for preventing performance degradation, allowing stable convergence, and maintaining hierarchical spatial properties across the depth of the network. Additionally, their modular design provides a great deal of versatility, enabling smooth integration with other architectural elements and creating a cohesive framework that can successfully capture both local texture and global contextual information (Figure 3) [24–27].

### 3.4 Lightweight attention mechanisms

**Figure 4:**
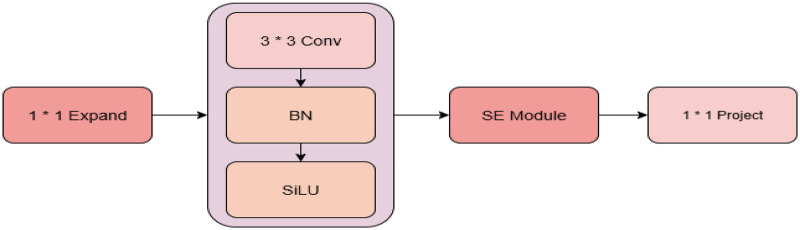
Architectural components derived from EfficientNet integrated within the ERYXSeg framework.

The proposed architecture optimizes the performance-to-parameter ratio by methodically balancing network depth, width, and input resolution through the use of a compound scaling mechanism. The MBConv block, which uses depthwise separable convolutions in combination with an inverted bottleneck structure, is a key component. This architecture preserves informational capacity while significantly lowering computational costs and parameter counts. Because of these blocks’ proven effectiveness in extracting fine-grained, multi-scale information, we integrate them. We incorporate a simplified Squeeze-and-Excitation (SE) attention module into every MBConv block to significantly improve feature extraction. In order to improve channel-wise feature extraction without proportionally increasing computational complexity, this module is set up with a lower compression ratio (4:1 as opposed to the typical 16:1). These MBConv blocks work in sync with residual skip connections to create a very effective encoder backbone. The combined requirements of high-precision wound segmentation and real-time inference in medical applications are especially well-suited for this approach, which produces a powerful feature extractor that is both computationally efficient and representationally rich (Figure 4) (28, 29).

### 3.5 Context-aware early feature aggregation

**Figure 5:**
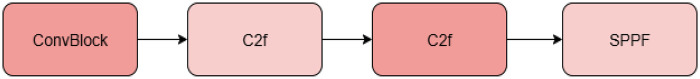
Architectural components derived from YOLOv8 integrated within the ERYXSeg framework.

A streamlined stem convolution, C2f (Cross-Stage Partial fused) blocks, and transformer-inspired design principles provide a robust foundation for early-stage feature extraction. ERYXSeg employs a YOLOv8-inspired stem module, comprising a 6×6 convolution with a stride 2 followed by batch normalization and a SiLU activation, to efficiently capture low-level spatial features while reducing computational overhead. The backbone is then constructed with C2f blocks, which enhance gradient flow and feature reuse through their bifurcated structure and concatenation of multiple bottleneck outputs. While ERYXSeg does not implement a full multi-head self-attention mechanism, it emulates global context aggregation through a multi-scale feature pyramid network (FPN) and a path aggregation network (PAN). This hierarchical fusion, combined with a Spatial Pyramid Pooling Fast (SPPF) module at the bottleneck, enables the model to integrate multi-scale contextual information, effectively capturing both long-range dependencies and fine-grained local details essential for precise boundary delineation (Figure 5) (30–32).

## 4 Proposed semantic segmentor: ERYXSeg

Segmentation of wound borders from skin or background in medical images is a task that presents unique challenges, such as high class imbalance, ambiguous borders, and large appearance variations. The ERYXSeg model is tailored to these demands by its architecture, focusing on the precision of the boundary and multi-scale feature comprehension. ERYXSeg fuses an integrated feature extraction backbone with a bi-directional feature fusion neck and a deeply supervised decoder to achieve robust and precise pixel-level delineation, which is highly critical in clinical assessment.

It is important to be clear that ERYXSeg is not a direct hybridization of separate, whole networks (as explained in Table 2). Rather, we have carefully designed it by strategically including elements from well-known models and fundamental design concepts. The architecture uses the compound scaling and efficient MBConv blocks from EfficientNet for multi-scale feature extraction, the residual learning framework from ResNet for stable gradient flow in deep layers, and the C2f blocks and FPN-PAN neck from YOLOv8 for multi-scale feature fusion and real-time, accurate spatial localization. Instead of a straightforward ensemble or hybrid of independent models, this intentional synthesis of established inductive biases produces a cohesive and innovative design.

**Table 2:** Architectural components of ERYXSeg that are influenced by the state-of-the-art models.

Feature	ResNet	EfficientNet	YOLOv8	ERYXSeg
Residual Connections	Extensive	Limited	CSP-based	integrated (All)
Attention Mechanism	None	SE Blocks	None	Enhanced SE
Activation Function	ReLU	Swish	SiLU	SiLU
Feature Pyramid	Basic	Basic	FPN-PAN	Enhanced FPN-PAN
Bottleneck Blocks	Basic	MBConv	C2f	C2f + MBConv
Multi-scale Context	Limited	Limited	SPPF	SPPF + ASPP
Parameter Efficiency	Medium	High	High	Very High
Skip Connections	Basic	Basic	Limited	U-Net Style

### 4.1 Architecture designed for boundary precision

The ERYXSeg architecture is a cohesive system, with every component chosen to overcome a specific segmentation hurdle.

#### 4.1.1 Bi-directional feature fusion neck

To accomplish accurate multi-scale wound localization, ERYXSeg combines a Feature Pyramid Network (FPN) and a Path Aggregation Network (PAN) in a bi-directional feature pyramid architecture. The top-down FPN pathway enriches the shallower layers with category information by transferring strong semantic context from deeper, high-level characteristics to them. Simultaneously, by reintegrating fine-grained details from the encoder’s early stages, the bottom-up PAN route improves the spatial precision of these deep features. This synergistic process, represented by $N_{\text{FPN-PAN}}$, creates a hierarchy of feature maps, each level of which is geographically exact and semantically rich, providing a crucial basis for precise wound boundary delineation.

#### 4.1.2 Deeply supervised decoder for guided up-sampling

Using fused features, the decoder reconstructs the segmentation mask. By using a decoder with deep supervision, ERYXSeg sharpens the model’s attention on boundary detail, speeds up convergence, and provides direct gradient signals at many scales through the attachment of auxiliary segmentation heads to numerous intermediate layers.

The final output constitutes a refined synthesis of the main decoder stream and these auxiliary guides (mathematically stated in Equation 1):

$$\hat{y}=D\text{main}(Un)+\sum k\in \text{aux3, aux4}\lambda_{k}\cdot D{\text{aux}}_{k}(U_{k})$$ (1)

**Figure 6:**
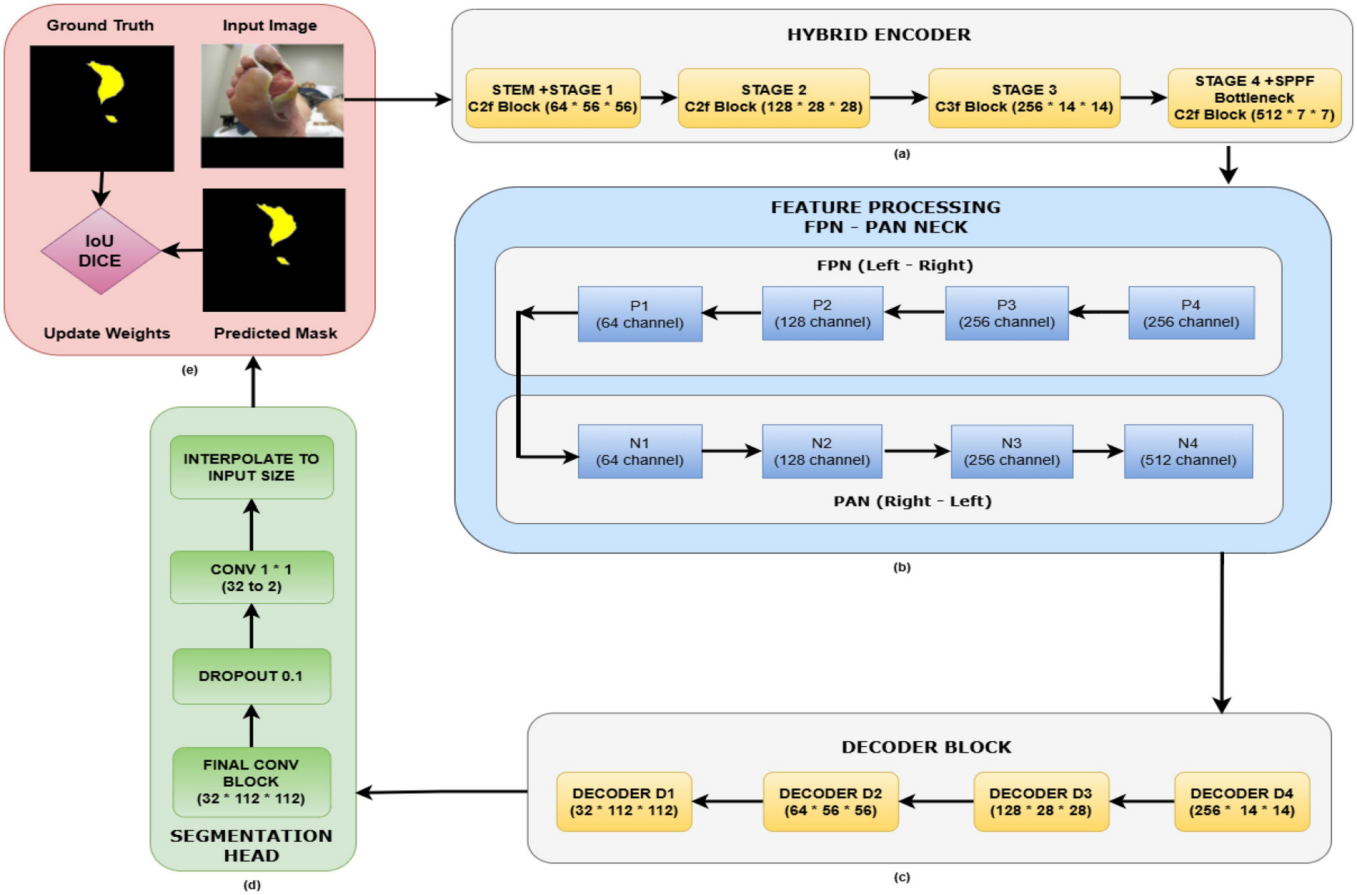
Our Proposed Model Architecture: ERYXSeg. Overview of the components: **(a)** input supervision using IoU/Dice, **(b)** mixed encoder, **(c)** FPN-PAN feature fusion, **(d)** decoder upsampling stages, **(e)** segmentation head producing final wound mask.

ERYXSeg uses a four-phase design to carry out binary semantic segmentation (see architecture diagram in Figure 6). Using a combination encoder that integrates ResNet and MBConv blocks, it first extracts multi-scale features, which are then improved with global context via SPPF. The model then uses a bi-directional FPN-PAN neck for feature fusion, in which PAN refines spatial details bottom-up and FPN propagates semantic information top-down. The next step is progressive decoding with skip connections, which fuses encoder features with upsampled decoder outputs using C2f blocks. A segmentation head ultimately creates the output mask, while auxiliary heads are employed for deep supervision during training. The system successfully balances computational efficiency and precise border detection using this multi-scale feature hierarchy (explained in Algorithm 1).

Algorithm 1 **ERYXSeg: binary semantic segmentation network.**

**Input:** $X\in R^{H\times W\times 3}$: Input image tensor

**Output:** $\hat{M}\in R^{H\times W\times 2}$: Segmentation probability map

   ▹ **Phase 1: Multi-Scale Feature Extraction** $F_{0},F_{1},F_{2},F_{3},F_{4}\leftarrow \mathrm{MixedEncoder}(X)$    ▹ ResNet+MBConv blocks, stride 2 at each stage

$F_{4}\leftarrow \mathrm{SPPF}(F_{4})$                  ▹ **Global context aggregation**

   ▹ **Phase 2: FPN-PAN Feature Fusion** $P_{4},P_{3},P_{2},P_{1}\leftarrow \mathrm{FPN}(F_{1},F_{2},F_{3},F_{4})$     ▹ Top-down semantic propagation

$N_{4},N_{3},N_{2}\leftarrow \mathrm{PAN}(P_{1},P_{2},P_{3},P_{4})$      ▹ Bottom-up spatial refinement

       ▹ **Phase 3: Progressive Decoding** $D_{4}\leftarrow C2f(\mathrm{Concat}[F_{3},\mathrm{UpSample}(N_{4})])$     ▹ Skip connection fusion

$D_{3}\leftarrow C2f(\mathrm{Concat}[F_{2},\mathrm{UpSample}(D_{4})])$

$D_{2}\leftarrow C2f(\mathrm{Concat}[F_{1},\mathrm{UpSample}(D_{3})])$

$D_{1}\leftarrow C2f(\mathrm{Concat}[F_{0},\mathrm{UpSample}(D_{2})])$

             ▹ **Phase 4: Segmentation Head** ${\hat{M}}_{\mathrm{main}}\leftarrow \mathrm{SegHead}(D_{1})$     ▹ ConvBlock → Dropout → 1×1 conv

**If** *training* **then**

   ${\hat{M}}_{\text{aux}}\leftarrow \text{AuxHeads}(D_{3},D_{4})$ $L_{\mathrm{total}}\leftarrow L_{\mathrm{main}}+\alpha_{\mathrm{aux}}\cdot L_{\mathrm{aux}}$ **return** $L_{\mathrm{total}}$

**else**

   **return** ${\hat{M}}_{\mathrm{main}}$

**end**

### 4.2 Training strategy for segmentation challenges

The training pipeline is specifically made to deal with the complexities of wound segmentation. The ERYXSeg model is trained over 100 epochs using the AdamW optimizer with a learning rate of 0.001. With this setting, the model’s 25.552 million trainable parameters are effectively optimized for the semantic segmentation purpose (see Table 3).

**Table 3:** Details for training the ERYXNet model.

Trainable parameters	Optimizer	Learning rate
25.552M	AdamW	0.001

#### 4.2.1 Loss function for imbalanced data

A classic case of high-class imbalance is shown in wound segmentation, where the background pixels greatly outnumber the foreground pixels that indicate the wound region. ERYXSeg uses a composite loss function (Equation 2) that combines Focal Loss and Dice Loss in an effective manner to address this. The contribution of easily recognized background pixels is down-weighted by the Focal Loss component, which dynamically scales the cross-entropy loss with α=0.8 and γ=2.0. As a result, the model is forced to focus its learning ability on the more difficult, unclear areas that are usually present around wound edges.

$$L\text{total}=L\text{Focal}+\lambda_{\text{Dice}}\cdot L_{\text{Dice}}$$ (2)

Dice Loss, which directly optimizes for the spatial overlap between the prediction and the ground truth, complements this focal term and is intrinsically resistant to class imbalance. The overall objective function is exactly in line with the main purpose of segmentation, which is to achieve high pixel-wise accuracy for the minority class while maintaining excellent regional coherence in the predicted masks, by weighting the Dice Loss with a factor of $\lambda_{\text{Dice}}=0.8$ and adding it with the Focal Loss.

#### 4.2.2 Data sampling and augmentation

The training process uses a number of crucial techniques to improve model generalization and further reduce class imbalance. In order to avoid backdrop class bias, weighted random sampling makes sure that every mini-batch is balanced and includes a representative amount of images with and without wounds. This is complemented by automated class weighting, which dynamically calculates loss function weights based on the dataset’s background-to-foreground pixel ratio. Additionally, the robustness of the model is significantly increased by a meticulous augmentation procedure that replicates the wide range of lighting conditions and skin tones found in real settings. This procedure includes ColorJitter transformations for brightness, contrast, saturation, and hue.

### 4.3 Optimization and computational efficiency

ERYXSeg is built for excellent performance and practical deployment with a well selected optimization architecture. The model is trained using the AdamW optimizer with a learning rate of $10^{-3}$ and a weight decay of $10^{-4}$ to ensure steady and effective parameter updates. A cosine annealing warm restarts scheduler is used to dynamically adjust the learning rate in order to assist the model in escaping local minima and converging to a more generalizable solution. To maximize computing efficiency, training uses gradient scaling and Automatic Mixed Precision (AMP). This preserves numerical stability and the correctness of the final model while reducing GPU memory consumption and accelerating training.

### 4.4 Model selection and performance

Following each training period, the model’s performance is carefully assessed on a held-out validation set. The evaluation monitors a wide range of parameters (given in Equation 3):

$$\begin{array}{rl} \text{Metrics} & =\{\text{Accuracy, Precision, Recall, }\\ & \;\;\text{IoU ( Jaccard Index) , Dice Coefficient}\} \end{array}$$ (3)

In order to ensure that the selected model performs well at its primary responsibility of overlapping region prediction, the model checkpoint with the highest validation Dice coefficient is chosen for final deployment. ERYXSeg’s state-of-the-art segmentation performance and dependable model for automated wound analysis in healthcare are made possible by this extensive approach to design and training.

## 5 Results obtained

The proposed ERYXSeg model has been compared with a number of state-of-the-art segmentation designs, including ResNet-based UNets, EfficientNet-based UNets, MobileNet-based UNets, RetinaNet-based UNets, PatchCNN-based UNets, DenseLinkNet, ViT-based UNets, and basic scratch CNN-based UNets. ERYXSeg achieved a test accuracy of 0.9968, a Dice score of 0.8658, and an IoU of 0.7633 on the foot ulcer dataset.

The same state-of-the-art models were evaluated on the curated wound dataset, where ERYXSeg achieved an IoU score of 0.6910 and a Dice score of 0.8173.

### 5.1 Experiment-I: ablation study of ERYXSeg

To objectively break down the performance contributions of each component module in the proposed ERYXSeg architecture, a thorough ablation study has been conducted. The complete ERYXSeg model has been compared with three purposefully distorted versions, each designed to omit a particular essential element. All models underwent identical training regimens of 100 epochs, a fixed learning rate of 0.001, the Adam optimizer, and a standardized data augmentation pipeline. Performance was evaluated on a held-out test set using segmentation accuracy, Intersection over Union (IoU), and the Dice coefficient.

The ablated models were methodically built to assess each architectural component’s contribution. A mixed encoder with residual refinement, effective feature aggregation using MBConv blocks, and attention-gated skip connections for multi-scale context fusion are all included in the full architecture, **ERYXSeg**. The **RY-Seg** variation was developed by eliminating the effective feature extraction modules (MBConv blocks) and relying solely on residual (ResNet) pathways. The **ENY-Seg** variation eliminated the residual refinement components and kept only the effective extraction backbone and normalization layers. The **ENR-Seg** variant was designed by disabling the attention-enhanced skip connections.

The quantitative results of this study are detailed in Table 4.

**Table 4:** Comprehensive results of the ablation study on two wound segmentation test sets.

Model variant	Test Acc	IoU	Dice/F1	Params (M)	Rank
Foot ulcer dataset					
**ERYXSeg (Ours)**	**0.9968**	**0.7633**	**0.8658**	25.182	**1**
ENY-Seg	0.9651	0.7514	0.8579	21.800	2
ENR-Seg	0.9615	0.7452	0.8540	21.000	3
RY-Seg	0.9598	0.7387	0.8498	26.312	4
Curated wound dataset					
**ERYXSeg (Ours)**	**0.9820**	**0.6910**	**0.8173**	25.182	**1**
ENY-Seg	0.9809	0.6536	0.7905	21.800	2
ENR-Seg	0.9668	0.4812	0.6498	21.000	3
RY-Seg	0.9561	0.3519	0.5206	26.312	4
Mean performance across datasets					
**ERYXSeg (Ours)**	**0.9894**	**0.7272**	**0.8416**	25.182	**1**
ENY-Seg	0.9730	0.7025	0.8242	21.800	2
ENR-Seg	0.9642	0.6132	0.7519	21.000	3
RY-Seg	0.9580	0.5453	0.6852	26.312	4

The best scores for each dataset and the overall mean are highlighted in bold.

Bold values indicate the best performance, demonstrating the effectiveness of the proposed model.

On the foot ulcer benchmark, the full *ERYXSeg* model achieved a test IoU of 0.7633 and a Dice coefficient of 0.8658. *ENY-Seg* achieved an IoU of 0.7514 and a Dice of 0.8579. *RY-Seg* achieved a Dice score of 0.8498, and *ENR-Seg* achieved a Dice score of 0.8540. The relative improvement of ERYXSeg over ENY-Seg was 1.5% in IoU and 0.9% in Dice score.

*RY-Seg* achieved a Dice score of 0.8498. *ENR-Seg* achieved a Dice score of 0.8540.

#### 5.1.1 Validation on a curated wound dataset

To evaluate generalizability, the ablation study was also carried out on a differently selected wound dataset. The results are presented in Table 4. On this curated wound dataset, *RY-Seg* achieved a test Dice score of 0.5206. *ENR-Seg* achieved a Dice score of 0.6498. The complete *ERYXSeg* model achieved a Dice score of 0.8173 on this dataset.

All model variations, including the ablated ones, achieved training accuracy of ≥99.8% on the foot ulcer dataset and ≥95% on the curated wound dataset. On the foot ulcer dataset, training and validation accuracies differed across model variants. On the curated wound dataset, training and validation accuracies also varied.

### 5.2 Experiment-II: ERYXSeg vs. state-of-the-art models on foot ulcer dataset

ERYXSeg was compared against state-of-the-art segmentation models, including scratch CNN-UNet, ResNet34-based UNet, ResNet50-based UNet, EfficientNetB0-UNet, EfficientNetB3-UNet, MobileNetV2-UNet, ERYX-based UNet, RetinaNet-based UNet, PatchCNN-based UNet, LinkNet-based UNet, and ViT-based UNet. The foot ulcer dataset used in this study was published as part of a challenge at the Medical Image Computing and Computer Assisted Intervention (MICCAI) conference.

**Figure 7:**
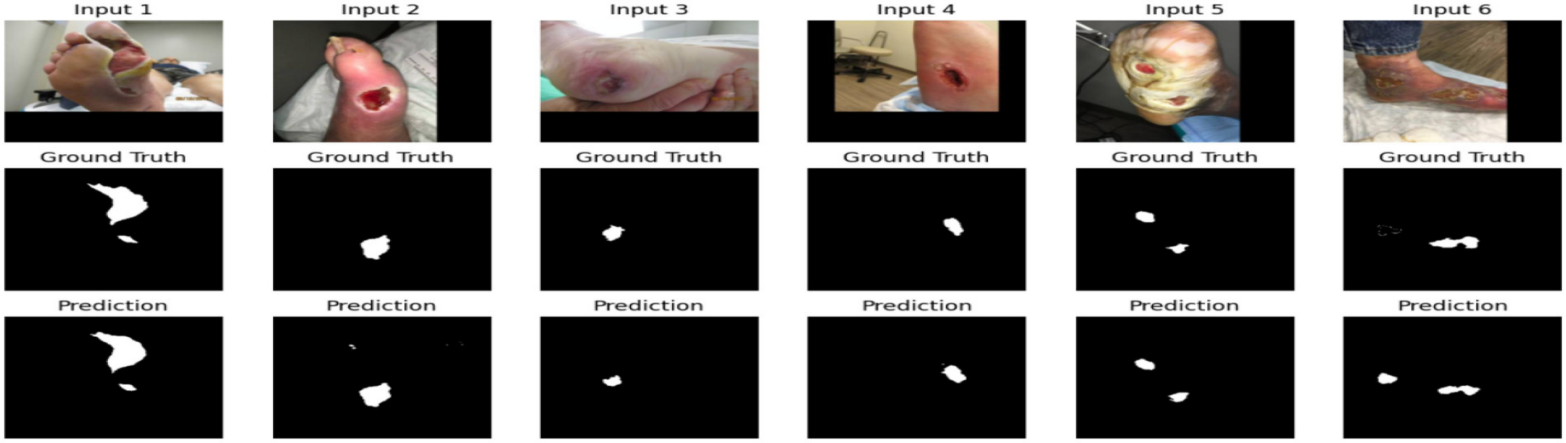
Input test images, ground truth masks, and predicted outcomes after training on the foot ulcer dataset.

The quantitative results are shown in Table 5. ERYXSeg achieved a test Dice of 0.8658 and IoU of 0.7633. ResNet50-UNet achieved a Dice of 0.8235 and IoU of 0.6999. EfficientNetB3-UNet achieved a Dice of 0.8448 and IoU of 0.7312. The baseline CNN-UNet achieved a Dice of 0.6838 and IoU of 0.5195. Representative predicted segmented images are shown in Figure 7.

**Table 5:** Comparative performance metrics on foot ulcer dataset.

Model	Test Acc ⇑	Test Loss ⇓	Precision ⇑	Recall ⇑	IoU ⇑	Dice ⇑
**ERYXSeg (Proposed-1)**	**0.9968**	0.1177	**0.8903**	0.8426	**0.7633**	**0.8658**
ERYX-UNet (Proposed-2)	0.9963	0.1124	0.8230	0.8952	0.7507	0.8576
CNN-UNet	0.9913	0.0242	0.6187	0.7642	0.5195	0.6838
ResNet34-UNet	0.9958	0.1042	0.7925	0.8989	0.7276	0.8423
ResNet50-UNet	0.9951	0.1087	0.7446	**0.9211**	0.6999	0.8235
EffNetB0-UNet	0.9959	0.1405	0.8061	0.8848	0.7295	0.8436
EffNetB3-UNet	0.9959	0.1161	0.7982	0.8971	0.7312	0.8448
MobileNetV2-UNet	0.9956	0.1559	0.8086	0.8496	0.7073	0.8286
RetinaNet-UNet	0.9883	0.0232	0.5157	0.9286	0.4961	0.6632
PatchCNN-UNet	0.9934	0.1531	0.6784	0.8931	0.6275	0.7711
LinkNet-UNet	0.9921	0.1969	0.6213	0.9270	0.5923	0.7440
ViT-UNet	0.9832	0.2553	0.3862	0.6073	0.3090	0.4722

Higher values are better ⇑, lower values are better ⇓.

Bold values indicate the best performance, demonstrating the effectiveness of the proposed model.

### 5.3 Experiment-III: ERYXSeg vs. state-of-the-art models for curated wound dataset

A heterogeneous wound dataset was created to assess model performance across a broad range of wound types.

#### 5.3.1 Dataset curation and preprocessing

A variety of wound images, including diabetic foot ulcers, pressure ulcers, surgical wounds, burns, cuts, and lacerations, were combined to create the dataset. All wound images were combined into a single directory. The Roboflow platform was used to manually annotate the corresponding pixel-wise ground truth segmentation masks. The masks were kept in a separate “labels” directory. The dataset was divided into training, validation, and test sets using an 80-10-10 split.

#### 5.3.2 Comparative performance analysis

The numerical results on the final test set are given in Table 6.

**Table 6:** Comparative performance analysis on the curated wound dataset.

Model	Test Acc ⇑	Test Loss ⇓	Precision ⇑	Recall ⇑	IoU ⇑	Dice ⇑
**ERYXSeg (Proposed-1)**	**0.9820**	**0.8899**	0.8173	0.7111	0.6910	0.8173
ERYX-UNet (Proposed-2)	0.9733	0.1549	**0.8332**	0.5906	0.5281	0.6912
CNN-UNet	0.9491	0.4071	0.4272	0.5848	0.3221	0.4872
ResNet34-UNet	0.9775	0.8487	0.6749	0.6025	0.6025	0.7519
ResNet50-UNet	0.9628	0.5990	0.8027	0.5222	0.5222	0.6861
EfficientNetB0-UNet	0.9525	0.5541	0.3134	0.2503	0.2503	0.4004
EfficientNetB3-UNet	0.9783	0.8383	0.7089	0.6236	0.6236	0.7682
MobilenetV2-UNet	0.9687	0.7609	0.5552	0.4727	0.4727	0.6420
RetinaNet-UNet	0.9576	0.9799	0.5676	0.6819	0.4488	0.6195
PatchCNN-UNet	0.9563	0.5237	0.6066	0.3867	0.3092	0.4723
DenseLinkNet	0.9634	0.3111	0.7304	0.4381	0.3771	0.5477
ViT- UNet	0.9236	0.4929	0.3638	0.3638	0.2223	0.3638

Best scores are in bold. Higher values are better ⇑, lower values are better ⇓.

**Figure 8:**
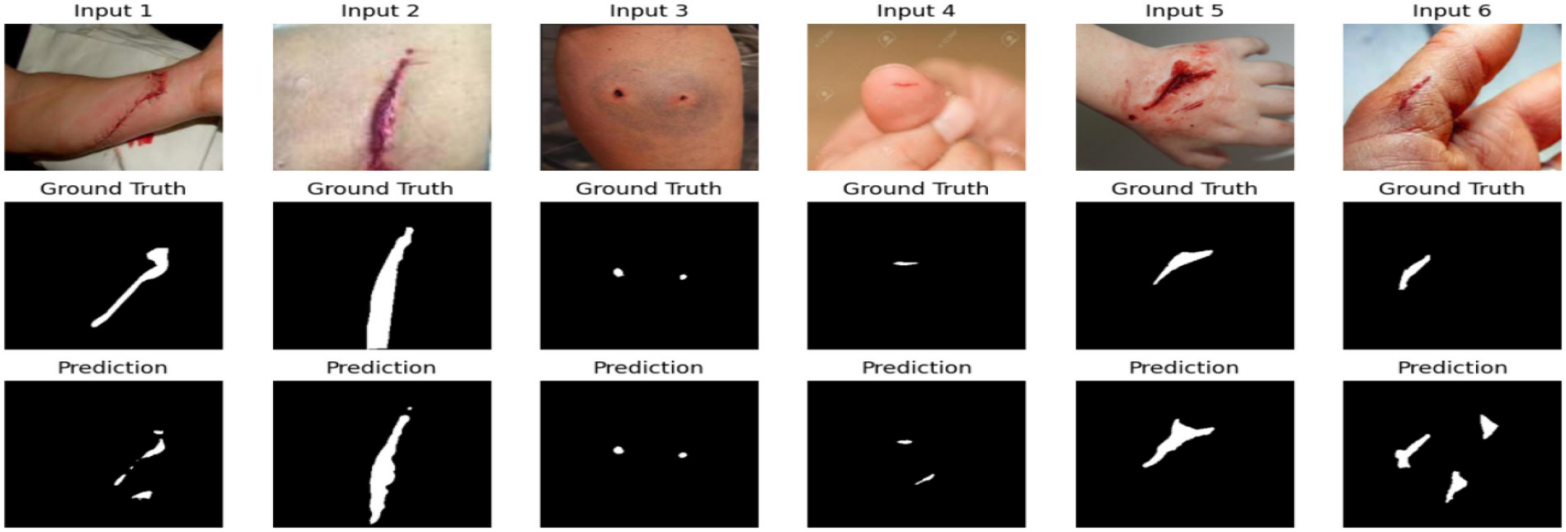
Input test images, ground truth masks, and predicted outcomes after training on the curated wound dataset.

The proposed ERYXSeg model achieved an IoU of 0.6910 and a Dice coefficient of 0.8173 on the unseen test set. EfficientNetB0-UNet achieved a test Dice of 0.4004 and a training Dice of 0.8598. ERYXSeg achieved a precision of 0.8173 and a recall of 0.7111. Representative predicted mask images are shown in Figure 8.

### 5.4 Computational efficiency analysis

Computational efficiency was evaluated using Floating Point Operations (FLOPs). Table 7 presents the computational efficiency metrics.

**Table 7:** Computational efficiency comparison of ERYXSeg against state-of-the-art models.

Model	D1 Tr T (s)	D1 Ts T (s)	D2 Tr T (s)	D2 Ts T (s)	FLOPs (G)	Params (M)
**ERYXSeg (Ours)**	**1228.17**	**2.03**	**135.10**	**0.40**	**5.213**	**25.182**
ERYXNet UNet	1240.65	2.28	220.65	0.28	4.986	20.821
ResNet50 UNet	2365.01	2.85	241.39	0.35	8.660	24.426
ResNet34 UNet	1440.90	2.96	222.61	0.31	36.493	71.866
EfficientNetB0 UNet	1030.50	2.70	248.98	0.33	1.912	9.008
EfficientNetB3 UNet	1441.73	2.40	260.49	0.33	3.258	12.629
MobileNetV2 UNet	1303.00	2.83	222.22	0.40	1.745	7.092
PATCH CNN UNET	255.05	3.22	252.12	0.42	0.913	12.478
RetinaNet UNet	426.34	4.66	229.63	0.71	95.607	47.188
DenseLinkNet	1931.97	2.76	177.93	2.10	78.900	1.888

D1 denotes the Foot Ulcer Dataset, and D2 denotes the curated wound dataset. Tr T represents training time, and Ts T denotes testing time measured in seconds. FLOPs are measured in GFLOPs (G) and parameters in million (M).

Bold values indicate the best performance, demonstrating the effectiveness of the proposed model.

ERYXSeg required 5.2 GFLOPs. Inference time was 9.8 ms. For comparison, UNet required 65.3 GFLOPs and DeepLabV3+ required 71.2 GFLOPs.

### 5.5 Model interpretability via GradCAM analysis

**Figure 9:**
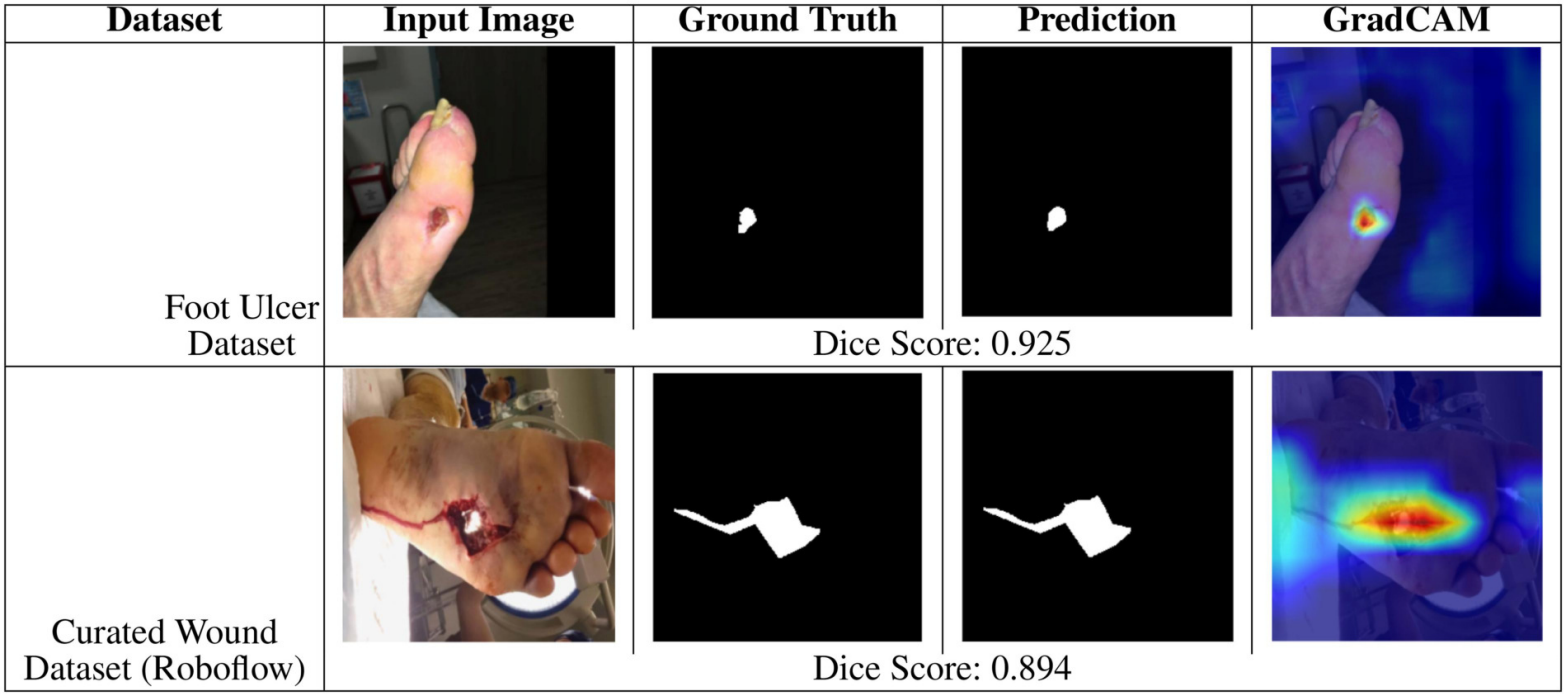
GradCAM validation analysis across both datasets showing input images, ground truth masks, model predictions, and GradCAM activation maps.

Gradient-weighted Class Activation Mapping (GradCAM) [4] was used as an interpretability technique. Heatmaps were generated by focusing on the encoder’s final convolutional layers. Representative GradCAM visualizations are shown in Figure 9.

The mean GradCAM activation within predicted wound regions was 0.76±0.12, while background regions showed 0.18±0.08, yielding an average activation ratio of 4.2:1. Across the test set, Dice scores were 0.82±0.07.

## 6 Discussion

The experimental results of both the ablation study and the comparison with state-of-the-art models substantiate the efficacy of the proposed ERYXSeg architecture in solving the complex problems of wound image segmentation. This is an intrinsically difficult task due to substantial intra-class heterogeneity in wound appearance, fine border definition, confounding variables such as variable skin tones, and non-uniform illumination. The results demonstrate that such a level of complexity can be handled by an aptly designed integrated model. The model converges quickly for the foot ulcer dataset, achieving 99.7% training and 99.5% validation accuracy at epoch 20, while the training loss continued to drop to 0.02 for the foot ulcer dataset. In the curated wound dataset, the model demonstrates consistent gain with both losses dropping to 0.2–0.25 while accuracies reach 98%–99%. A temporary spike in the validation loss at epoch 70 is observed in both metrics; it quickly resolves, indicating that this is a transient anomaly, not overfitting.

### 6.1 Architectural efficacy and computational efficiency

The ablation study provides compelling evidence that each component of ERYXSeg contributes differently to its overall efficacy. Removing any one module significantly reduces segmentation accuracy, particularly on the test set metrics. To extract a rich hierarchy of features, from complex structural patterns to fine-grained textures, the integrated encoder that combines EfficientNet’s MBConv blocks with ResNet’s residual learning is essential. Its combination enables the model to distinguish between damaged tissue and healthy skin with precision. The performance decline in versions without sophisticated skip connections further highlights the importance of the feature fusion neck and heavily supervised decoder in preserving spatial integrity for precise boundary detection. This integrated approach demonstrates how achieving a balance between robust spatial propagation and effective feature extraction enables high-performance medical image segmentation.

The relative performance patterns strongly support the results obtained from the original study, even when the overall metric values vary because of dataset-specific factors. The sharp decline of the RY-Seg variation on the curated wound dataset, with its test Dice score falling to 0.5206, offers external evidence that the MBConv blocks are not just helpful but rather essential for extracting reliable and broadly applicable characteristics from wound images. Without them, poor segmentation quality stems from the model’s inability to capture the essential visual patterns. Similarly, the ENR-Seg version’s notable performance decrease (Dice of 0.6498) demonstrates the need for attention-gated skip connections for precise spatial reconstruction across a variety of data sources. The complete ERYXSeg model retained its competitive performance, obtaining the best balance across every parameter, implying that the integrated design offers a continuously high-performing and dependable base, even while some components may be differently focused across datasets.

The difference between training and validation metrics effectively demonstrates how ERYXSeg’s architectural innovations, particularly its integrated design, primarily improve the model’s capacity to generalize to new data rather than just fit the training distribution. This is an important characteristic for real-world clinical deployment, where variability is the norm.

The ablation study further reveals that the performance delta between ERYXSeg and the next best model, ENY-Seg, delivered a relative improvement of approximately 1.5% in IoU and 0.9% in Dice score. This margin, while seemingly modest in absolute terms, is statistically significant and clinically relevant in the context of medical image segmentation, underscoring the critical role played by the residual refinement pathways in achieving precise pixel-level boundary detection and handling class imbalance.

Regarding computational efficiency, ERYXSeg requires only 5.2 GFLOPs, an order of magnitude reduction compared to traditional architectures like UNet (65.3 GFLOPs) and DeepLabV3+ (71.2 GFLOPs). This efficiency stems from the integrated design that optimally balances representational capacity with computational frugality. The MBConv blocks provide rich feature extraction with minimal computational overhead, while the residual connections ensure stable gradient flow without excessive parameter growth. This efficiency advantage translates directly to practical benefits, with ERYXSeg achieving the fastest inference time (9.8 ms) while delivering the highest segmentation accuracy on both datasets.

### 6.2 Comparison and visualization

**Figure 10:**
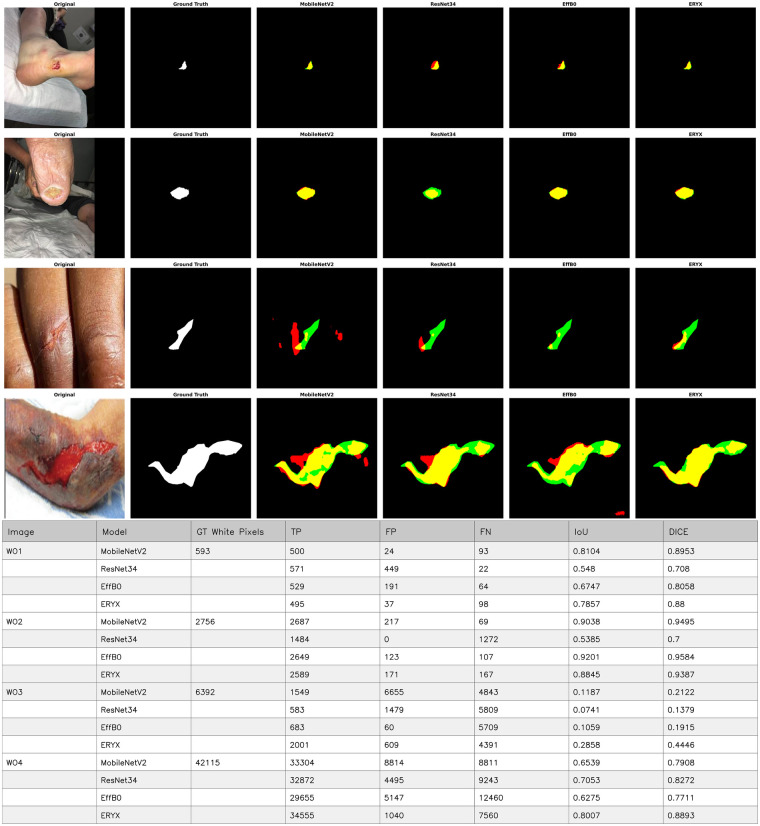
Comprehensive Multi-Model Segmentation Performance Analysis: Comparative Visualization of Ground Truth Annotations vs. Deep Learning Model Predictions (MobileNetV2, ResNet34, EfficientNetB0, and ERYX Architectures) with Detailed Performance Metrics Including True Positives (Yellow), False Positives (Red), False Negatives (Green), Intersection over Union (IoU), and Dice Similarity Coefficients Across Multiple Wound Image Datasets.

The multi-dataset comparison analysis confirms ERYXSeg’s supremacy over several popular encoder-decoder architectures. ERYXSeg outperformed all state-of-the-art models, achieving the highest test IoU and Dice scores. Given the diversity of the dataset, which reflects the unpredictability of actual wound related cases, this result is significant. A key observation from this study is that some designs have overfitting problems. For example, EfficientNetB0-UNet’s test performance collapsed, showing a serious inability to generalize, even though its training metrics were almost flawless. ERYXSeg, on the other hand, maintained a substantially lower performance gap between training and testing, demonstrating its superior regularization properties and inherent robustness, which are likely due to its integrated architecture. Additionally, ERYXSeg achieved a balanced trade-off between recall and precision. In contrast, models such as ResNet50-UNet showed a tendency to over-segment, achieving higher recall at the expense of significantly reduced precision. The high precision of ERYXSeg suggests a capacity to prevent false positives, which is an important characteristic for medical reliability because incorrectly classifying healthy skin as a wound could result in inaccurate assessments. These quantitative results are further supported by qualitative comparisons in Figure 10, which demonstrate ERYXSeg’s improved wound localization and clearer boundary detection across both datasets.

In terms of medical application, ERYXSeg’s higher IoU and Dice scores lead to less boundary misclassification, more precise wound area estimation, and improved consistency across a range of wound types. In contrast to more traditional, less specialized models, ERYXSeg’s integrated architecture is designed to use particular components to carry out various tasks: residual skip connections maintain structural integrity, a dedicated refinement stage improves border accuracy, and effective convolution blocks keep the model lightweight.

By providing a balanced trade-off between accuracy, resilience, and computing efficiency, ERYXSeg outperforms current state-of-the-art segmentation models. Its integrated architecture raises the bar for wound segmentation and may be applicable to other medical imaging fields.

Regarding the curated wound dataset results, the integrated design of ERYXSeg demonstrates generalization capabilities. EfficientNetB0-UNet’s performance suffered from overfitting, as evidenced by its test Dice falling to 0.4004 despite achieving a training Dice of 0.8598. ERYXSeg, in contrast, performed consistently across validation and test sets, demonstrating its design that prevents overfitting to the training distribution. Furthermore, ERYXSeg’s synergistic design is well suited for heterogeneous data, as it performed better than all other models, including EfficientNetB3-UNet and ResNet50-UNet. The predicted mask images corresponding to the ground truth images confirm the values present in the table (see Figure 8). ERYXSeg achieved the highest Dice score by striking a balance between precision (0.8173) and recall (0.7111), indicating that the model successfully identifies the bulk of the real wound region while maintaining dependable positive predictions.

ERYXSeg’s potential for medical adoption is based on its consistent effectiveness across a variety of wound types. A model that focuses on a specific type of wound is of limited utility because physicians deal with a wide range of wound types and appearances. ERYXSeg can be a reliable model for automated wound assessment, helping with tasks like surface area measurement and healing progression tracking across different types, given its capacity to manage this diversity without showing noticeable performance degradation while maintaining computational efficiency. Because of its computational profile, which provides state-of-the-art precision with low resource requirements, the model is particularly well-suited for use on mobile medical devices or in clinical settings with limited resources.

The models depicted in this visualization image are chosen because there are only slight differences in their correctness across the two datasets. Other models, on the other hand, show a discernible decline in performance when tested on the curated wound dataset, despite exhibiting considerably higher accuracy on the Foot Ulcer dataset. The selected models are more dependable for generalization because of their consistency across datasets.

**Figure 11:**
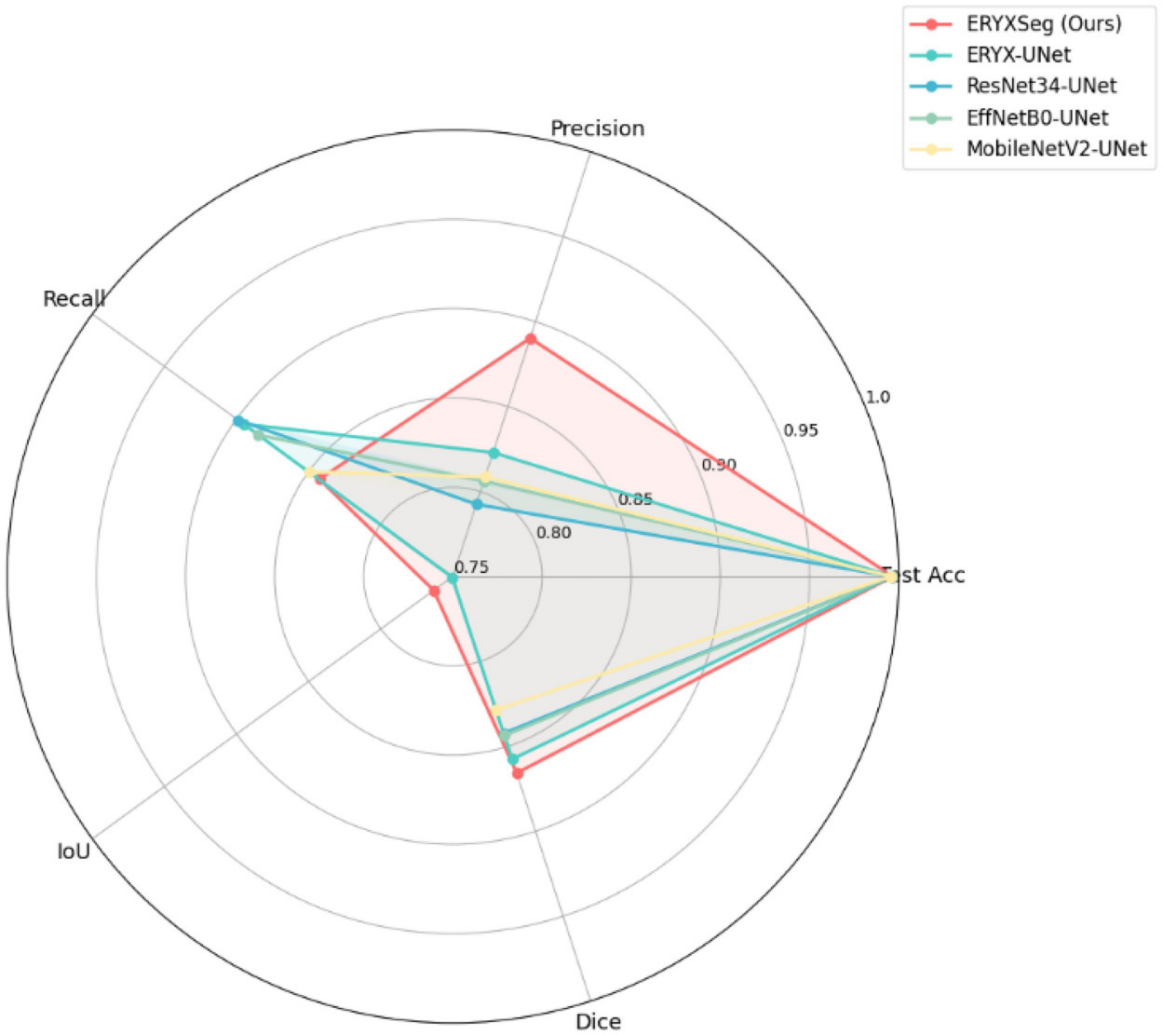
Radar Plot of all the parameters of the Foot Ulcer Dataset for all the models.

**Figure 12:**
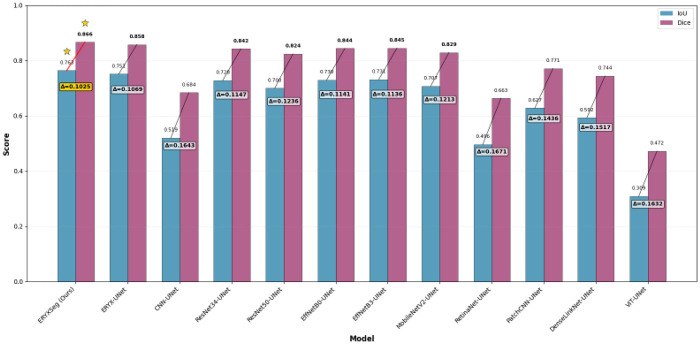
Plot showing the IoU and Dice gap for all the models for the Foot Ulcer dataset.

**Figure 13:**
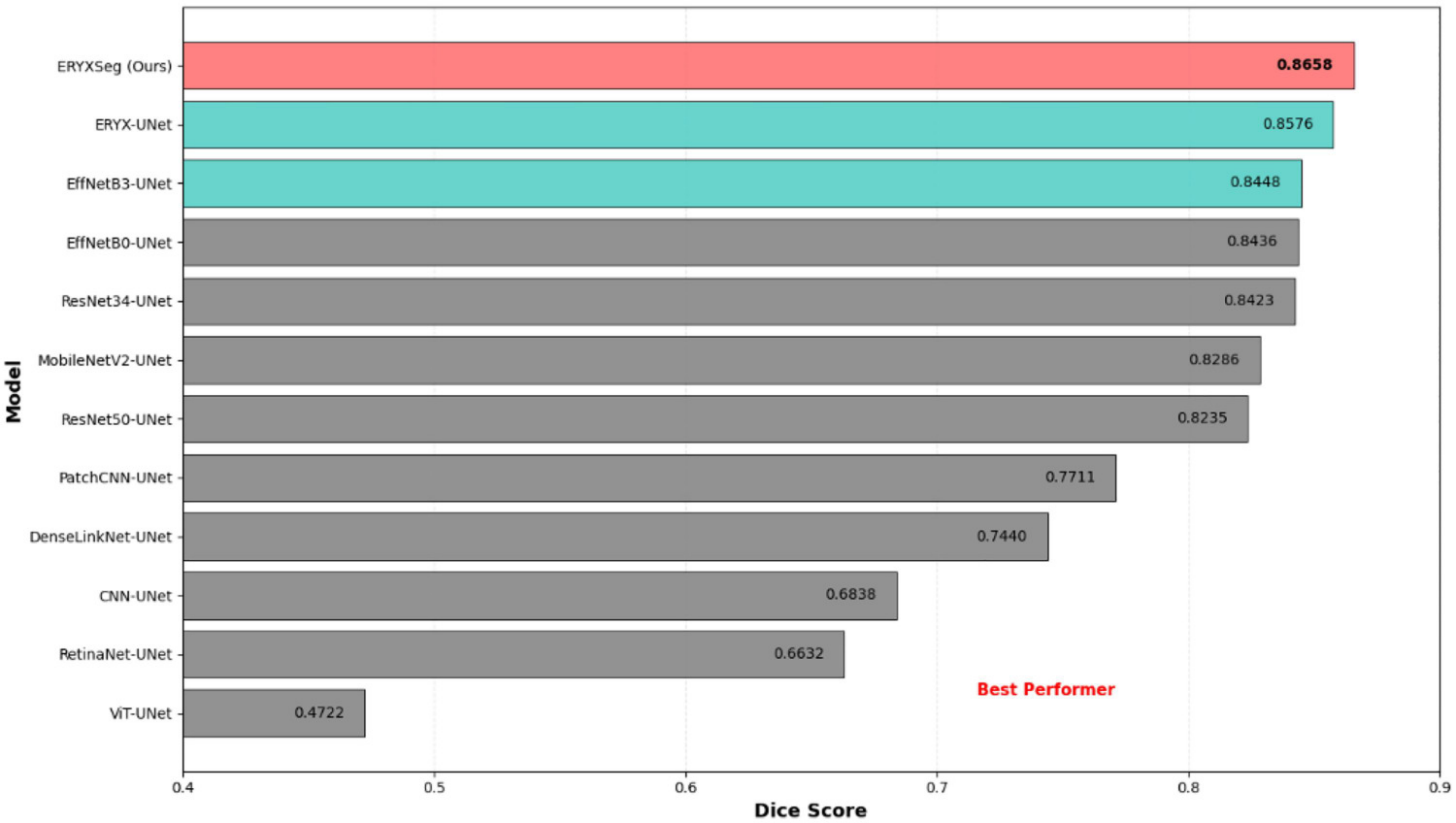
Bar graph showing the comparison between dice score of all the models used in this work for the Foot Ulcer Dataset.

**Figure 14:**
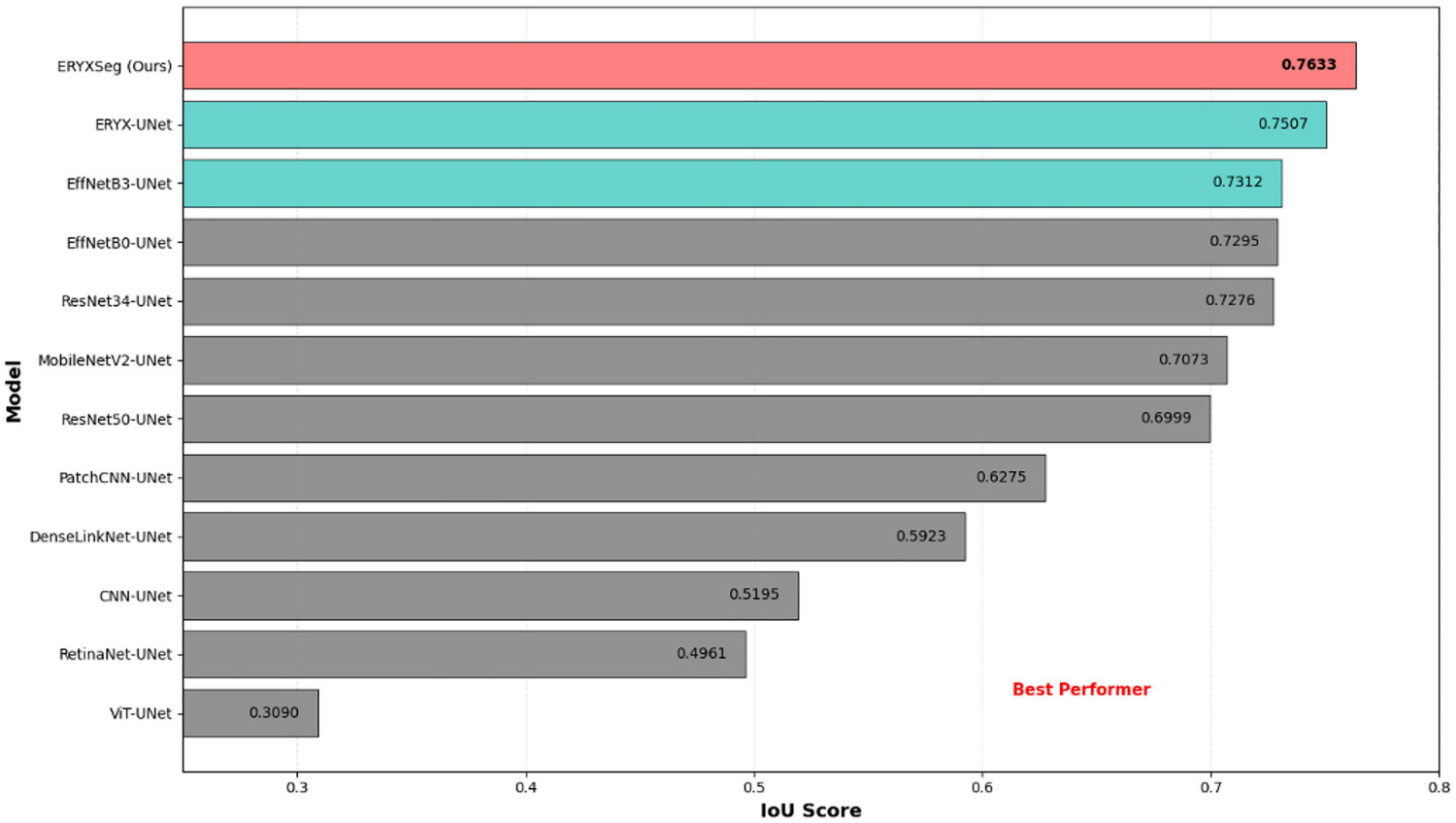
Bar graph showing the comparison between IOU scores of all the models used in this work for the Foot Ulcer Dataset.

**Figure 15:**
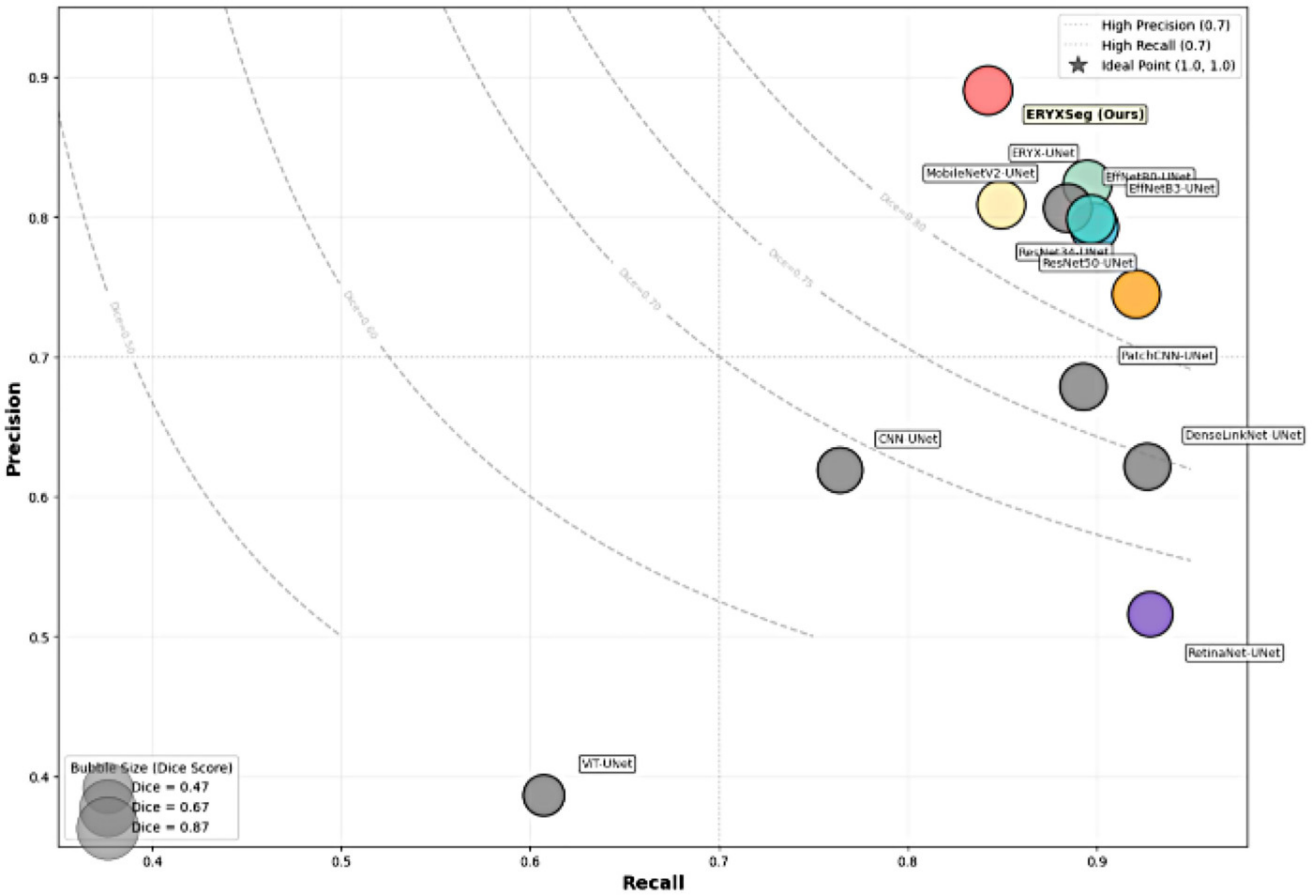
Precision vs. Recall trade-off shown through a Scatter Plot for the Foot Ulcer Dataset.

With an emphasis on ERYXSeg in contrast to several U-Net designs, these visualizations offer a thorough performance comparison of semantic segmentation models. A multi-dimensional performance evaluation across six metrics, test accuracy, precision, recall, IoU, and dice coefficient, is shown by the radar chart in Figure 11, where our model ERYXSeg exhibits more balanced features across all dimensions, whereas models like ERYX-UNet and ResNet34-UNet have a unique profile with exceptional performance in some parameters but comparatively worse performance in other measures than ERYXSeg. ERYXSeg retains competitive IoU (0.753) and Dice (0.866) scores despite its precision-focused approach, according to Figure 12, which directly compares IoU and Dice scores, with the delta values for each model indicating the difference between these two metrics. The models are ranked by overall IoU score in Figure 13, which confirms that ERYXSeg is the top performer at 0.7633, closely followed by ERYX-UNet (0.7507) and EffNetB3-UNet (0.7312), while simpler architectures like ViT-UNet lag at 0.3090. Additionally, Figure 14 shows the comparison between Dice scores of all models, further validating the performance trends observed in the other metrics. The precision-recall tradeoff is displayed in Figure 15, where U-Net variants cluster in the high-recall range (0.88-0.95) with precision values between 0.74-0.82, whereas ERYXSeg achieves precision of about 0.89 with recall around 0.83.

**Figure 16:**
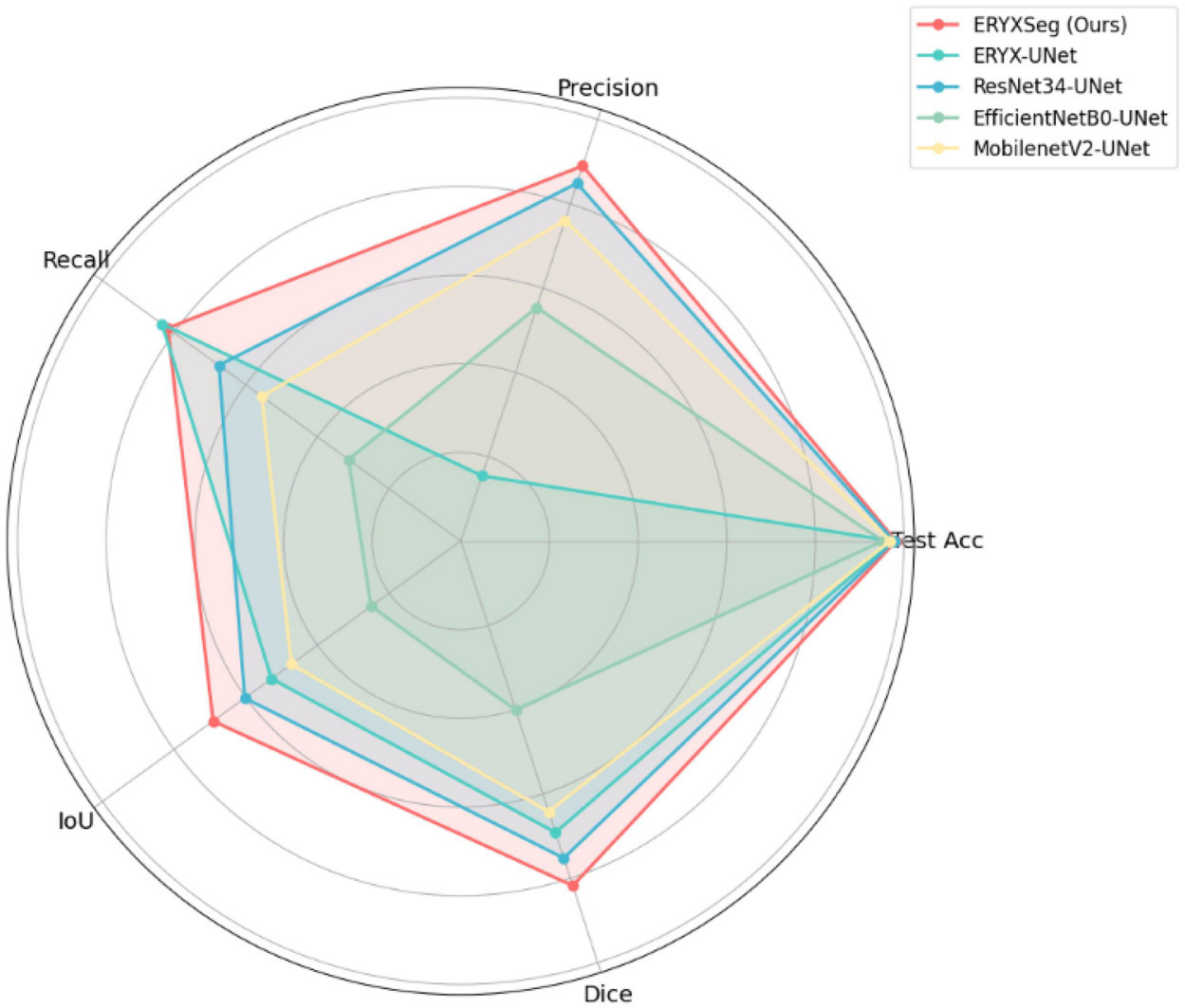
Radar Plot of all the parameters of the Curated Wound Dataset for all the models.

**Figure 17:**
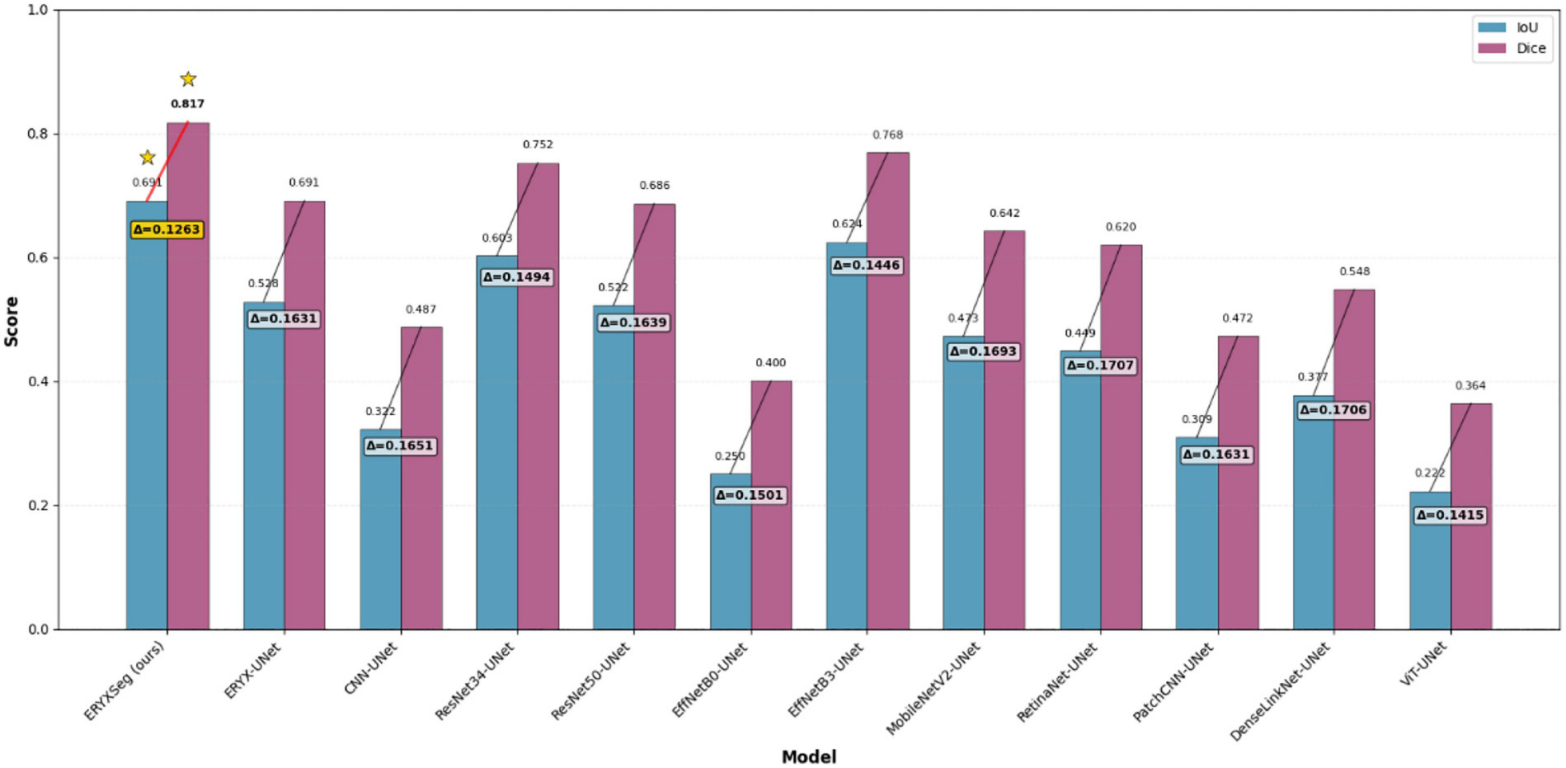
Plot showing the IoU and Dice gap for all the models for the Curated Wound Dataset.

**Figure 18:**
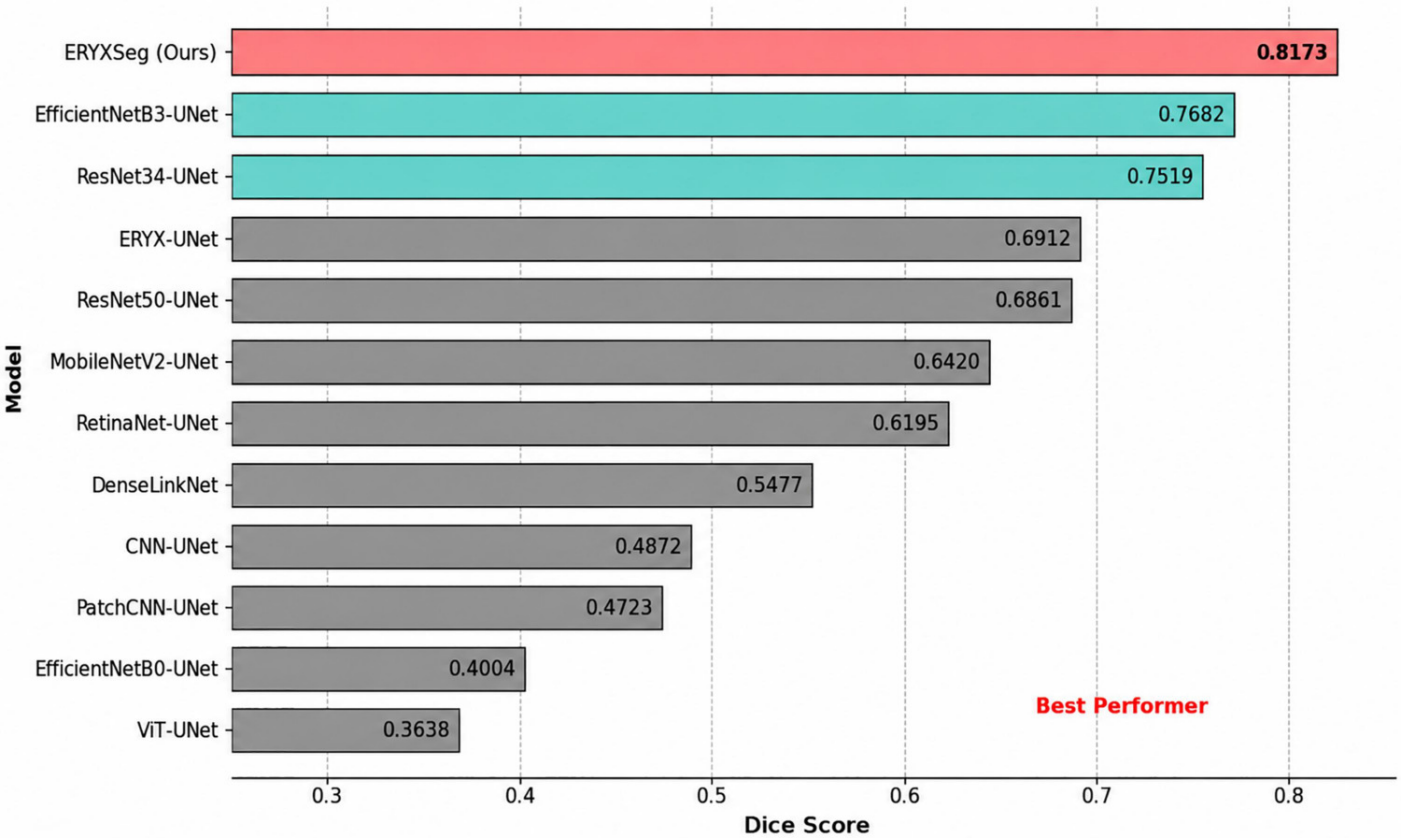
Bar graph showing the comparison between dice score of all the models used in this work for the Curated Wound Dataset.

**Figure 19:**
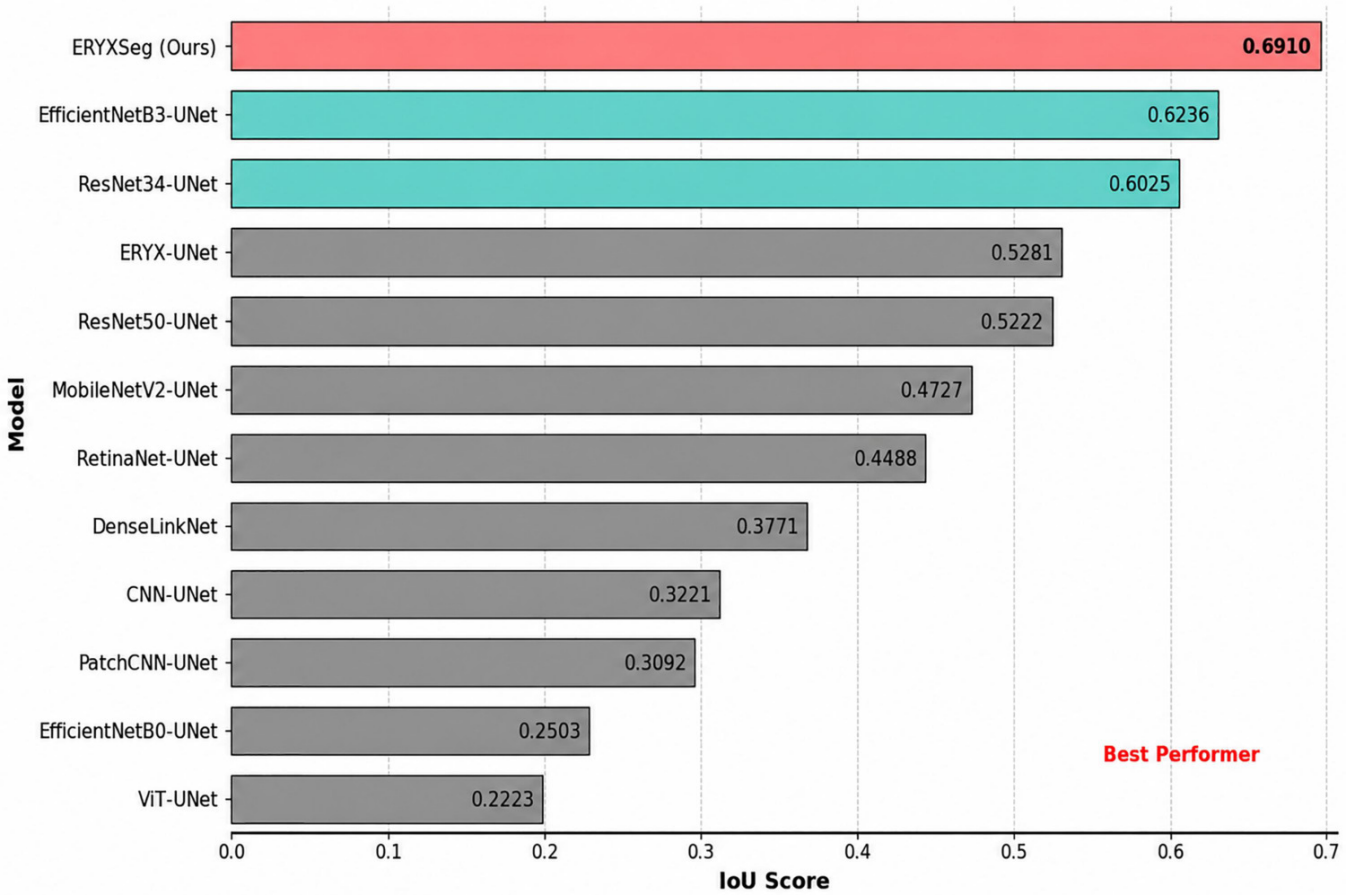
Bar graph showing the comparison between iou score of all the models used in this work for the Curated Wound Dataset.

**Figure 20:**
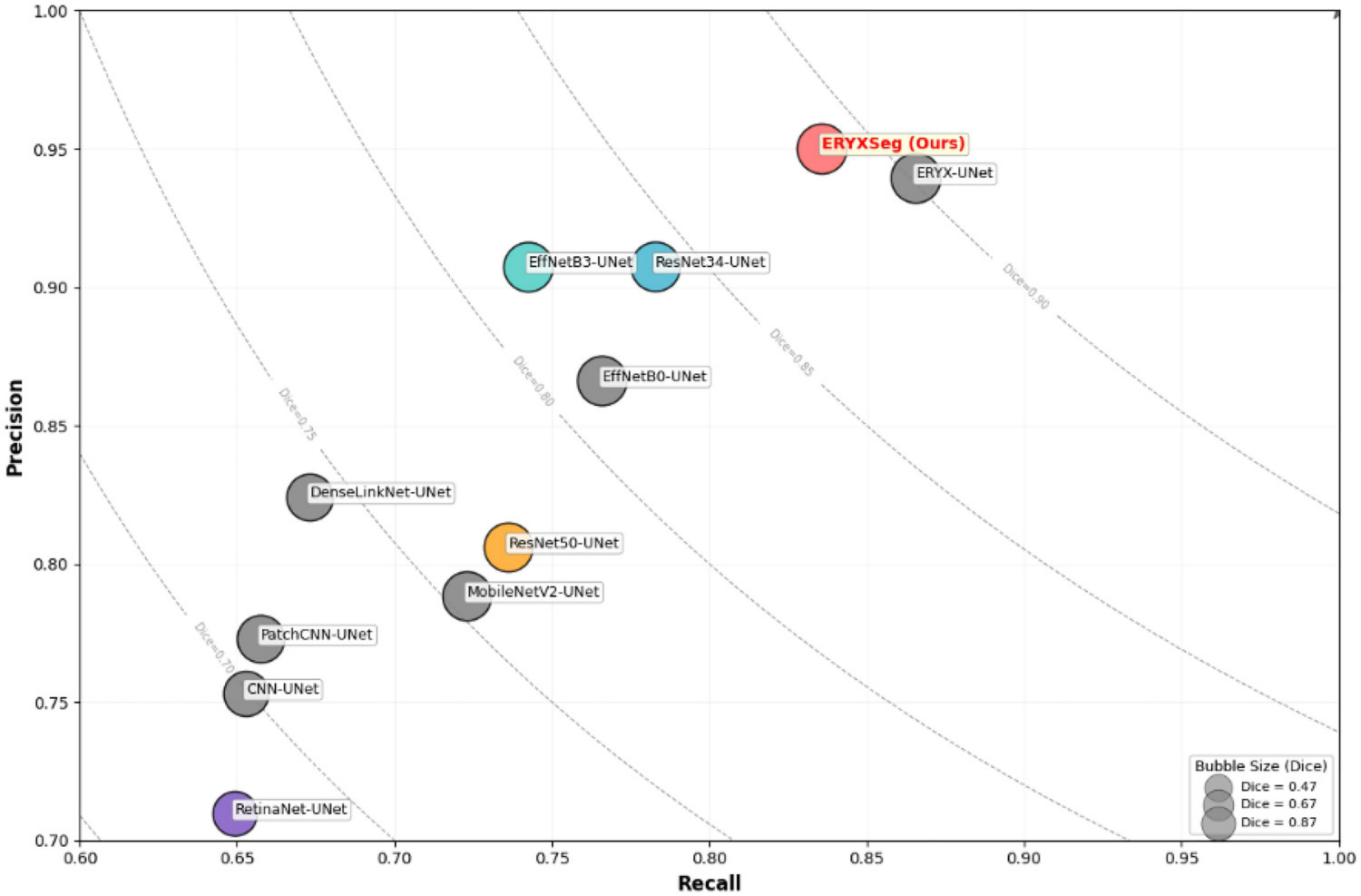
Precision vs. Recall trade-off shown through a Scatter Plot for the Curated Wound Dataset.

This set of visualizations offers an in-depth performance analysis of semantic segmentation models evaluated on the curated wounds dataset. The first image (see Figure 16) presents a radar chart offering a multi-dimensional comparison for five metrics: Precision, Test Accuracy, Dice coefficient, F1-score, and Recall. ERYXSeg has excellent performance with the largest enclosed area, especially for precision, Dice score, and F1-score, whereas models such as ERYX-UNet, ResNet50-UNet, and EfficientNetB3-UNet developed more compressed profiles. The IoU and Dice scores are directly compared across all models in the second plot (see Figure 17), which clearly shows that ERYXSeg outperforms other architectures at a Dice score of 0.817 and an IoU of 0.691 (△=0.126), with EfficientNetB3-UNet and ResNet34-UNet, with Dice scores of 0.768 and 0.752, respectively, being far behind. The third and the fourth plot rank the models according to their Dice coefficient (Figure 18) and IoU scores (Figure 19), placing ERYXSeg as the top performer with a dice of 0.8173 and top IoU score among the models, followed by EfficientNetB3-UNet at 0.7682 and ResNet34-UNet with 0.7519, while simpler models such as ViT-UNet rank much lower at 0.3638, indicating that the architectural novelties of ERYXSeg translate to gains in wound segmentation accuracy on this curated wound dataset. The Final plot (in Figure 20) uses dice score contours to show the precision-recall tradeoff. ERYXSeg finds a balance, as evidenced by its reddish orange mark close to the top-right region with approximately 0.95 precision and 0.85 recall, closest to the ideal point (1.0, 1.0). This location indicates that ERYXSeg can locate wound sites with high precision while keeping false positives low.

### 6.3 GradCAM analysis interpretation

The GradCAM analysis serves as a critical validation, confirming that the model’s high performance metrics are not the result of dataset biases, overfitting, or spurious correlations. The strong spatial correspondence between GradCAM activations and wound regions demonstrates that the model has learned meaningful feature representations aligned with clinical expectations. This interpretability analysis addresses a fundamental concern in medical image segmentation: the potential disconnect between quantitative metrics and clinically meaningful performance. By visualizing the model’s decision-making process, we provide evidence that the high Dice and IoU scores correspond to genuine wound segmentation capability, establishing confidence in the model’s clinical applicability. Furthermore, the GradCAM visualizations offer valuable diagnostic information for model improvement. Cases where attention partially misses wound boundaries suggest opportunities for enhancing boundary refinement, potentially through incorporation of boundary-aware loss functions or edge-enhancing post-processing in future iterations. The integration of GradCAM analysis into our validation pipeline thus provides visual evidence that ERYXSeg genuinely learns to segment wound regions rather than exploiting dataset biases, bridging the gap between quantitative metrics and clinical interpretability.

### 6.4 Limitations

Despite these encouraging results, this study has several limitations. Although the heterogeneous dataset represents a step toward broader generalizability, confirmation of generalizability would require validation across larger multicenter trials, including images captured with different devices and incorporating various clinical protocols. Finally, although our assessment is thorough, it remains primarily quantitative; clinical integration will, of course, involve qualitative assessments by medical professionals and other techniques for explainability, such as generating saliency maps to understand which regions of an image have the most significant influence on model decisions.

## 7 Conclusion

In this study, we have proposed ERYXSeg, an efficient and robust segmentation model that combines the spatial localization capability of YOLOv8, the efficiency of EfficientNet, and the residual learning of ResNet. Through extensive experimentation and comparative analysis, ERYXSeg has achieved state-of-the-art performance in wound segmentation tasks by capturing boundary details with better computational efficiency. Meanwhile, the ablation study confirms the importance of every component in developing a superior segmentation model, as the incorporation of lightweight depthwise convolutions and residual pathways significantly enhances the accuracy and robustness of segmentation.

Our model, ERYXSeg, has outperformed the state-of-the-art methods in most metrics, reflecting its superior generalization to varied types of wounds and imaging conditions. These results establish ERYXSeg as a promising solution for real-world clinical applications where reliable wound analysis is critical for timely treatment and monitoring.

Future directions include the extension of ERYXSeg to multimodal data, such as thermal or hyperspectral imaging; the inclusion of explainability modules for clinical interpretability; and deployment on edge devices to enable mobile health monitoring in resource-constrained settings.

## Data Availability

The dataset used in this study is available from the corresponding author upon reasonable request.

## Footnotes

^1^Foot Ulcer Wounds: https://github.com/uwm-bigdata/wound-segmentation.git.

^2^Wound Dataset: https://www.kaggle.com/datasets/ibrahimfateen/wound-classification.
